# Impact of obesity on outcomes after surgical stabilization of multiple rib fractures: Evidence from the US nationwide inpatient sample

**DOI:** 10.1371/journal.pone.0299256

**Published:** 2024-02-29

**Authors:** Yang-Fan Liu, Te-Li Chen, Ching-Hsueh Tseng, Jen-Yu Wang, Wen-Ching Wang

**Affiliations:** 1 Department of Thoracic Surgery, Hsinchu MacKay Memorial Hospital, Hsinchu, Taiwan; 2 Department of Life Science & Institute of Molecular and Cellular Biology, National Tsing Hua University, Hsinchu, Taiwan; 3 International Intercollegiate Ph.D. Programme, National Tsing Hua University, Hsinchu, Taiwan; 4 Department of Emergency, Hsinchu MacKay Memorial Hospital, Hsinchu, Taiwan; E-Da Cancer Hospital, TAIWAN

## Abstract

**Background:**

Obesity is a global health issue with increasing prevalence. Surgical procedures, such as surgical stabilization of rib fractures (SSRF), may be affected by obesity-related complications. The objective of the study is to investigate the effects of obesity on SSRF outcomes in multiple rib fractures.

**Methods:**

This retrospective study analyzed data from adults aged ≥ 20 years in the Nationwide Inpatient Sample (NIS) database diagnosed with multiple rib fractures who underwent SSRF between 2005 and 2018. It investigated the relationship between obesity and in-patient outcomes, such as discharge status, length of stay (LOS), in-hospital mortality, hospital costs, and adverse events using logistic and linear regression analyses.

**Results:**

Analysis of data from 1,754 patients (morbidly obese: 87; obese: 106; normal weight: 1,561) revealed that morbid obesity was associated with longer LOS (aBeta = 0.07, 95% CI: 0.06, 0.07), higher hospital costs (aBeta = 47.35, 95% CI: 38.55, 56.14), increased risks of adverse events (aOR = 1.63, 95% CI: 1.02, 2.61), hemorrhage/need for transfusion (aOR = 1.77, 95% CI: 1.12, 2.79) and mechanical ventilation ≥ 96 hours (aOR = 2.14, 95% CI: 1.28, 3.58) compared to normal weight patients. Among patients with flail chest, morbid obesity was significantly associated with tracheostomy (aOR = 2.13, 95% CI: 1.05, 4.32), ARDS/respiratory failure (aOR = 2.01, 95% CI: 1.09, 3.70), and mechanical ventilation ≥ 96 hours (aOR = 2.80, 95% CI: 1.47, 5.32). In contrast, morbid obesity had no significant associations with these adverse respiratory outcomes among patients without a flail chest (p > 0.05).

**Conclusions:**

Morbid obesity is associated with adverse outcomes following SSRF for multiple rib fractures, especially for flail chest patients.

## Introduction

Obesity is defined by the World Health Organization (WHO) as having a body mass index (BMI) of 30 or higher. The WHO has reported a significant increase in global obesity rates over the last five decades. The National Health and Nutrition Examination Survey (NHANES) of the United States revealed an age-adjusted obesity prevalence of 42.4% for 2017‒2018, with no differences across age groups or genders [1]. Apart from being associated with an increased risk of various chronic diseases, obesity is also associated with specific challenges during surgical procedures. This includes prolonged operation durations [2–4], anesthesia-related challenges [5, 6], and an elevated risk of complications, particularly in orthopedic surgeries [7, 8]. Thoracic trauma involves a wide range of injuries associated with significant morbidity and mortality, accounting for approximately 35% of trauma-associated deaths in the United States [9]. These injuries are usually blunt or penetrating trauma occurring as a result of automobile accidents, sports injuries, falls, violent acts such as gun shootings or stabbings, and explosions. Among the outcomes, rib fractures, particularly those caused by substantial forces, are prevalent [10]. Severe cases often present multiple rib fractures, some accompanied by a flail chest. Management of these injuries spans both operative and nonoperative methods.

Surgical stabilization of rib fractures (SSRF) is a treatment employed to address severe or multiple rib fractures using surgical techniques such as plating or wiring. The conventional approach for SSRF, which necessitates a sizable incision, has been debated due to the substantial tissue damage and associated complications. Nevertheless, advances in materials and technologies have refined the SSRF procedure, significantly mitigating associated complications [11]. Recent studies suggest that SSRF can effectively alleviate pain, reduce postoperative opioid usage, decrease ventilation days, shorten intensive care unit stays, and reduce overall hospital length of stay (LOS) for patients with major trauma [12, 13]. The influence of obesity on surgical outcomes is well-documented, with obese patients typically facing worse results than their non-obese counterparts. While it is logical to anticipate that obesity may lead to complications and poorer outcomes in the context of SSRF, solid evidence relating obesity to outcomes is limited.

The objective of this study was to examine the relationship between obesity and short-term outcomes of SSRF in patients with multiple rib fractures in a comprehensive nationwide inpatient database.

## Materials and methods

### Study design and data source

This is a retrospective, observational, and population-based study. The study extracted admission data from the US Nationwide Inpatient Sample (NIS), the largest all-payer, continuous inpatient care repository in the United States (US), encompassing approximately 8 million hospital stays annually [14]. Administered by the Healthcare Cost and Utilization Project (HCUP) of the US National Institutes of Health (NIH), this database is a valuable source of hospitalized patients’ primary and secondary diagnoses, procedures, discharge status, demographic features, insurance status, and various hospital characteristics such as region, hospital scale, and location/teaching status. It draws its data from a diverse set of about 1,050 hospitals located in 44 states across the US, making it a representative 20% stratified sample of the nation’s community hospitals.

### Ethics statement

The data for this study were obtained through a formal request to the Online Hcup Central Distributor, which serves as the database administrator. To ensure compliance with data usage regulations, we adhered to the NIS data-use agreement with Hcup. As a secondary data analysis utilizing information from the NIS, the study did not involve direct engagement with patients or the general public.

### Study population

Data of adults aged ≥20 years who were hospitalized in the US due to multiple rib fractures and subsequently underwent SSRF between the years 2005 and 2018. The accuracy of all recorded diagnoses and procedures was confirmed using the appropriate International Classification of Diseases, Ninth and Tenth Revisions (ICD-9 and ICD-10) diagnostic/procedure codes. Patients with only one rib fracture, or without complete data on discharge destination, concurrent brain or abdominal injury, underweight (BMI<19 kg/m^2^), hospital costs, in-hospital death, or database weight values were excluded. The patients were classified into three distinct groups based on the WHO criteria for BMI/obesity for further comparison: morbidly obese (BMI>40 kg/m^2^), obese (≥30 and ≤40 kg/m^2^), and normal weight (<30 kg/m^2^).

### Study variables and outcome measures

This study evaluated the following outcomes: 1) hospital LOS; 2) unfavorable discharge, defined as discharge to long-term care facilities; 3) in-hospital mortality; 4) total hospital costs; and 5) adverse outcomes, including tracheostomy, pneumonia, surgical site infection (SSI), sepsis, hemorrhage/need for transfusion, cerebrovascular accident (CVA), acute myocardial infarction (AMI), venous thromboembolism (VTE), acute kidney injury (AKI), respiratory failure/ acute respiratory distress syndrome (ARDS), and mechanical ventilation ≥ 96 hours, all identified through ICD codes.

### Covariates

The patients’ characteristics encompassed various factors such as age, sex, race, household income level, insurance status, smoking status, and major comorbidities, including conditions like diabetes, hypertension, chronic kidney disease (CKD), ischemic heart disease, congestive heart failure, atrial fibrillation, anemia, chronic obstructive pulmonary disease (COPD), cerebrovascular disease, peripheral vascular disease, severe liver disease, coagulopathy, and any malignancy. These comorbidities were identified using the appropriate ICD-9 and ICD-10 diagnostic codes. Additionally, the study also considered the number of days from admission to surgery and the type of fracture (with or without flail chest) as relevant characteristics. To provide a comprehensive analysis, hospital-related characteristics such as hospital region, hospital bed size, and location/teaching status, were also extracted from the database for all included patients. The ICD codes used in this study are summarized in S1 Table in S1 File. Hospital bed size was categorized as small, medium, and large, and the criteria also included hospitals’ region, location, and teaching status, which are summarized in S2 Table in S1 File.

### Statistical analysis

The NIS covers 20% of the US inpatient admissions annually. Weighted samples (TRENDWT before 2011; DISCWT after 2012), clusters (HOSPID), and stratum (NIS_STRATUM) were used to obtain national estimates. SAS provides analysis of sample survey data using the SURVEY procedure. Descriptive statistics, presented as weighted percentages (%) or mean and standard error (SE), were used to summarize the data. Categorical data were analyzed using the PROC SURVEYFREQ statement, while continuous data were analyzed with the PROC SURVEYREG statement. 95% confidence intervals (CIs) and Odds ratios (ORs) were presented for the dichotomized outcomes using logistic regression analysis; and estimates using linear regression analysis. Variables with significant differences between the two comparison groups were adjusted before entering into multivariable regression models. All statistical tests were two-sided, and statistical significance was determined by p-values less than 0.05 (p < 0.05). The statistical software package SAS version 9.4 (SAS Institute Inc., Cary, NC, USA) was used to perform all the analyses.

## Results

### Study population selection

**Fig 1:**
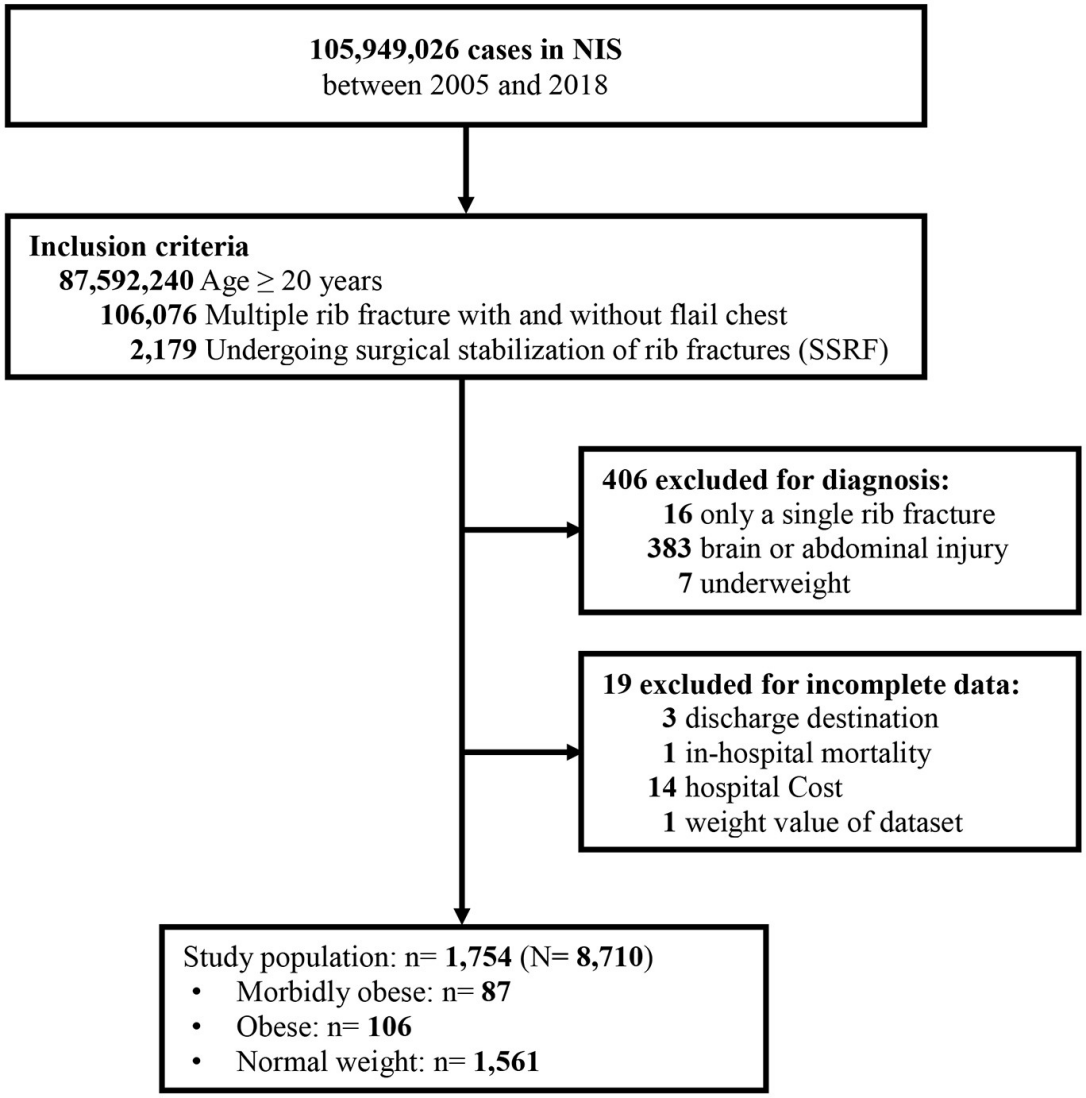
Flow chart of sample selection.

Fig 1 provides a flow diagram depicting the selection process of the study population. From 2005 to 2018, a total of 2,179 adults with multiple rib fractures with and without flail chest undergoing SSRF in the NIS database were identified. Patients with only a single rib fracture (n = 16), brain or abdominal injury (n = 383), or underweight (n = 7) and those with incomplete data (n = 19) were excluded. Thus, 1,754 patients were included in the study, representing 8,710 hospitalized adults across the US. Of this cohort, 87 were categorized as morbidly obese, 106 as obese, and 1,561 as normal weight (Fig 1).

### Population characteristics

Table 1 delineates the demographics and characteristics of the participants. The mean age was 56.7 ± 0.3 years, with males constituting 72.5% of the population. Private insurers, inclusive of Health Maintenance Organizations (HMOs), account for 46.8% of the total. Additionally, 32.9% of patients were smokers, 38.3% had hypertension, 94.7% had no pneumothorax, and 94.1% had an injury severity score (ISS) of severe. The mean time interval from admission to surgery was 3.3 ± 0.1 days. Flail chest was diagnosed in 36.4% of the patients, while 63.6% did not have it. Of all the patients, the majority stay in hospitals with a large bed size (75.6%), mostly in urban teaching hospitals (79.8%), with the highest proportion of patients coming from the West US region (30.1%). When stratified by BMI, morbidly obese patients were typically younger with a higher percentage of congestive heart failure (p < 0.05). In addition, obese patients had higher percentages of smoking, diabetes, hypertension, CKD, and anemia (all p < 0.05).

**Table 1:** Characteristics of the study population.

	Multiple rib fracture w/o flail chest undergoing SSRF				
	Total	Morbidly obese (BMI > 40 kg/m^2^)	Obese (≤ 30 BMI < 40 kg/m^2^)	Normal weight (19≤ BMI <30 kg/m^2^)	p-value
	(n = 1754)	(n = 87)	(n = 106)	(n = 1561)	
**Age, mean years (SD)**	56.7 ± 0.3	54.3 ± 0.8	59.7 ± 0.6	56.7 ± 0.4	**0.015**
20–39	229 (13.0)	10 (11.5)	11 (10.3)	208 (13.3)	0.134
40–59	765 (43.6)	47 (54.1)	43 (40.6)	675 (43.2)	
60+	760 (43.4)	30 (34.4)	52 (49.1)	678 (43.5)	
**Sex**					0.096
Male	1273 (72.5)	59 (67.6)	70 (65.8)	1144 (73.3)	
Female	481 (27.5)	28 (32.4)	36 (34.2)	417 (26.7)	
**Insurance status**					0.131
Medicare/Medicaid	626 (35.9)	25 (28.8)	45 (42.8)	556 (35.9)	
Private including HMO	819 (46.8)	49 (56.5)	45 (43.0)	725 (46.5)	
Self-pay/no-charge/other	302 (17.3)	13 (14.7)	15 (14.2)	274 (17.6)	
Missing	7	0	1	6	
**Household income** ^a^					0.089
Quartile1	414 (24.2)	18 (20.8)	28 (26.6)	368 (24.2)	
Quartile2	453 (26.4)	32 (37.9)	30 (28.6)	391 (25.6)	
Quartile3	446 (26.2)	21 (24.9)	29 (27.7)	396 (26.2)	
Quartile4	396 (23.2)	14 (16.5)	18 (17.1)	364 (24.0)	
Missing	45	2	1	42	
**Smoking**					**0.002**
No	1179 (67.1)	50 (57.8)	59 (55.4)	1070 (68.4)	
Yes	575 (32.9)	37 (42.2)	47 (44.6)	491 (31.6)	
**Major comorbidities**					
Diabetes	278 (15.8)	27 (31.3)	35 (33.0)	216 (13.8)	**<0.001**
Hypertension	671 (38.3)	47 (54.0)	65 (61.1)	559 (35.9)	**<0.001**
CKD	68 (3.9)	4 (4.6)	9 (8.4)	55 (3.5)	**0.027**
Ischemic heart disease	175 (10.0)	9 (10.5)	14 (13.1)	152 (9.8)	0.489
Congestive heart failure	75 (4.3)	8 (9.3)	7 (6.6)	60 (3.9)	**0.011**
Atrial fibrillation	148 (8.5)	11 (12.7)	13 (12.3)	124 (8.0)	0.085
Anemia	53 (3.0)	2 (2.3)	7 (6.6)	44 (2.8)	**0.038**
COPD	167 (9.5)	13 (14.7)	12 (11.3)	142 (9.0)	0.130
Cerebrovascular disease	30 (1.7)	1 (1.2)	2 (1.9)	27 (1.7)	0.887
Peripheral vascular disease	49 (2.8)	2 (2.3)	2 (1.9)	45 (2.9)	0.792
Severe liver disease	8 (0.5)	0 (0.0)	0 (0.0)	8 (0.5)	NA
Rheumatic disease	28 (1.6)	3 (3.5)	2 (1.9)	23 (1.5)	0.327
Coagulopathy	42 (2.4)	1 (1.2)	5 (4.7)	36 (2.3)	0.215
Any malignancy	20 (1.1)	1 (1.2)	1 (0.9)	18 (1.2)	0.964
**Days from admission to surgery**	3.3 ± 0.1	3.7 ± 0.3	3.1 ± 0.2	3.3 ± 0.1	**0.001**
**Fracture type**					**0.028**
Without flail chest	1115 (63.6)	46 (52.8)	74 (69.8)	995 (63.8)	
With flail chest	639 (36.4)	41 (47.2)	32 (30.2)	566 (36.2)	
**Pneumothorax**					0.392
No	1661 (94.7)	81 (93.1)	103 (97.3)	1477 (94.6)	
Yes	93 (5.3)	6 (6.9)	3 (2.7)	84 (5.4)	
**ISS** ^c^					0.670
Non-severe	104 (5.9)	6 (6.9)	8 (7.6)	90 (5.8)	
Severe	1650 (94.1)	81 (93.1)	98 (92.4)	1471 (94.2)	
**Hospital bed size** ^b^					0.146
Small	99 (5.7)	3 (3.5)	8 (7.8)	88 (5.6)	
Medium	324 (18.7)	10 (11.6)	18 (17.5)	296 (19.2)	
Large	1313 (75.6)	74 (84.9)	77 (74.7)	1162 (75.2)	
Missing	18	0	3	15	
**Hospital location/teaching status**					**0.008**
Rural	35 (2.1)	2 (2.3)	3 (2.8)	30 (2.0)	
Urban nonteaching	318 (18.1)	24 (27.2)	24 (23.3)	270 (17.3)	
Urban teaching	1383 (79.8)	61 (70.5)	76 (73.9)	1246 (80.7)	
Missing	18	0	3	15	
**Hospital region**					0.112
Northeast	264 (15.2)	13 (14.9)	11 (10.4)	240 (15.5)	
South	453 (25.7)	28 (32.2)	38 (35.8)	387 (24.7)	
Midwest	511 (29.0)	22 (25.3)	28 (26.3)	461 (29.4)	
West	526 (30.1)	24 (27.6)	29 (27.6)	473 (30.4)	

Continuous variables are presented as mean ± SE; categorical variables are presented as unweighted counts (weighted percentage).

P-value < 0.05 is shown in bold.

Abbreviations: SSRF, surgical stabilization of rib fractures; HMO, Health Maintenance Organization, CKD, chronic kidney disease; COPD, chronic obstruction pulmonary disease; ISS, injury severity score; SD, standard deviation; w/o, with/without; NA, not applicable.

^a^ Income level was defined per HCUP quartile classification of estimated median household income of residents in patients’ ZIP Codes.

^b^ Information of hospital bed size is documented in S2 Table in S1 File.

^c^ ISS ≥ 9 is defined as severe.

### Perioperative outcomes

Perioperative outcomes are summarized in Table 2. The average LOS was 11.6 ± 0.2 days with hospital costs averaging 193.8 ± 4.4 thousand US dollars. The percentages of unfavorable discharge, in-hospital mortality, and any complication in the total study population were 30.7%, 0.9%, and 51.8%, respectively. The morbidly obese group had a higher mean LOS and increased incidences of adverse events, hemorrhage/need for transfusion, ARDS/respiratory failure, and mechanical ventilation (≥ 96 hours) (all p < 0.05).

**Table 2:** Perioperative outcomes.

Characteristic	Multiple rib fracture w/o flail chest undergoing SSRF				
	Total	Morbidly obese (BMI > 40 kg/m^2^)	Obese (≤ 30 BMI < 40 kg/m^2^)	Normal weight (19≤ BMI <30 kg/m^2^)	p-value
	(n = 1754)	(n = 87)	(n = 106)	(n = 1561)	
**LOS, day** ^a^	11.6 ± 0.2	14.2 ± 0.6	10.3 ± 0.3	11.6 ± 0.2	**0.004**
**Unfavorable discharge** ^a^					0.298
No	1203 (69.3)	54 (62.4)	72 (68.1)	1077 (69.7)	
Yes	535 (30.7)	32 (37.6)	34 (31.9)	469 (30.3)	
**In-hospital mortality**					NA
No	1738 (99.1)	86 (98.8)	106 (100.0)	1546 (99.0)	
Yes	16 (0.9)	1 (1.2)	0 (0.0)	15 (1.0)	
**Hospital costs (per 1000 dollars)**	193.8 ± 4.4	239.4 ± 8.5	174.8 ± 4.3	192.5 ± 4.6	**0.021**
**Adverse events, any**	909 (51.8)	55 (63.7)	58 (54.8)	796 (50.9)	**0.029**
Tracheostomy	150 (8.5)	11 (12.9)	5 (4.5)	134 (8.5)	0.076
Pneumonia	250 (14.2)	16 (18.6)	16 (15.1)	218 (13.9)	0.427
SSI	22 (1.2)	2 (2.4)	0 (0.0)	20 (1.3)	NA
Sepsis	142 (8.1)	10 (11.7)	9 (8.6)	123 (7.8)	0.379
Hemorrhage/need for transfusion	519 (29.6)	36 (41.8)	34 (32.3)	449 (28.7)	**0.017**
VTE	79 (4.4)	7 (8.0)	6 (5.6)	66 (4.2)	0.137
AKI	184 (10.5)	14 (16.1)	13 (12.2)	157 (10.0)	0.126
AMI	12 (0.7)	0 (0.0)	1 (0.9)	11 (0.7)	NA
CVA	426 (24.2)	27 (31.4)	26 (24.5)	373 (23.7)	0.207
ARDS/respiratory failure	512 (29.1)	35 (40.6)	30 (28.2)	447 (28.5)	**0.023**
Mechanical ventilation ≥ 96 hours	287 (16.3)	26 (30.2)	15 (14.0)	246 (15.7)	**<0.001**

Continuous variables are presented as mean ± SE; categorical variables are presented as unweighted counts (weighted percentage).

P-value < 0.05 is shown in bold.

Abbreviations: LOS, length of stay; SSI, surgical site infection; VTE, venous thromboembolism; AKI, acute kidney injury; AMI, acute myocardial infarction; CVA, cerebrovascular accident; ARDS, acute respiratory distress syndrome; w/o, with/without; NA, not applicable

^a^ Excluding patients who died in hospitals.

### Associations between obesity status and perioperative in-hospital outcomes

Table 3 shows the links between obesity status and perioperative outcomes. When adjusted for possible confounders, patients with morbid obesity experienced a significantly longer LOS (adjusted Beta (aBeta), 0.07, 95% CI: 0.06, 0.07) and higher hospital costs (aBeta, 47.35, 95% CI: 38.55, 56.14) compared to normal weight patients. Moreover, morbid obesity was significantly associated with increased occurrences of adverse events (adjusted odds ratio (aOR), 1.63, 95% CI: 1.02, 2.61), hemorrhage/need for transfusion (aOR, 1.77, 95% CI: 1.12, 2.79) and mechanical ventilation (≥ 96 hours) (aOR, 2.14, 95% CI: 1.28, 3.58) compared to normal weight. Full analytic models are documented in S3 and S4 Tables in S1 File.

**Table 3:** Associations between obesity status and perioperative outcomes.

Outcomes	Obesity status ^g^	Univariate		Multivariable	
		Beta/OR (95%CI)	p-value	aBeta/aOR (95%CI)	p-value
**LOS, days** ^a^ ^b^					
	**Morbidly obese**	**0.07 (0.07, 0.07)**	**<0.001**	**0.07 (0.06, 0.07)**	**<0.001**
	**Obese**	**0.02 (0.00, 0.03)**	**0.006**	-0.02 (-0.03, 0.00)	0.077
	**Normal weight**	Ref.		Ref.	
**Unfavorable discharge** ^a^ ^c^					
	**Morbidly obese**	1.39 (0.91, 2.11)	0.125	1.43 (0.86, 2.36)	0.167
	**Obese**	1.08 (0.72, 1.62)	0.701	0.90 (0.56, 1.43)	0.650
	**Normal weight**	Ref.		Ref.	
**In-hospital mortality** ^d^					
	**Morbidly obese**	1.22 (0.16, 9.31)	0.850	0.79 (0.16, 3.82)	0.773
	**Obese**	NA		NA	
	**Normal weight**	Ref.		Ref.	
**Hospital costs** ^e^					
	**Morbidly obese**	**46.85 (7.26, 86.44)**	**0.020**	**47.35 (38.55, 56.14)**	**<0.001**
	**Obese**	-17.72 (-42.76, 7.32)	0.165	**-7.22 (-12.25, -2.19)**	**0.005**
	**Normal weight**	Ref.		Ref.	
**Adverse events, any** ^f^					
	**Morbidly obese**	**1.69 (1.14, 2.52)**	**0.009**	**1.63 (1.02, 2.61)**	**0.040**
	**Obese**	1.17 (0.81, 1.69)	0.409	1.13 (0.75, 1.69)	0.560
	**Normal weight**	Ref.		Ref.	
**Tracheostomy** ^f^					
	**Morbidly obese**	1.60 (0.86, 2.98)	0.140	1.32 (0.69, 2.55)	0.400
	**Obese**	0.51 (0.23, 1.16)	0.107	0.44 (0.17, 1.11)	0.082
	**Normal weight**	Ref.		Ref.	
**Pneumonia** ^f^					
	**Morbidly obese**	1.41 (0.82, 2.44)	0.216	1.15 (0.60, 2.20)	0.670
	**Obese**	1.10 (0.66, 1.83)	0.716	1.04 (0.60, 1.81)	0.892
	**Normal weight**	Ref.		Ref.	
**SSI** ^f^					
	**Morbidly obese**	1.89 (0.43, 8.25)	0.395	2.19 (0.39, 12.19)	0.369
	**Obese**	NA		NA	
	**Normal weight**	Ref.		Ref.	
**Sepsis** ^f^					
	**Morbidly obese**	1.56 (0.79, 3.06)	0.198	1.39 (0.63, 3.06)	0.420
	**Obese**	1.10 (0.60, 2.03)	0.754	1.16 (0.60, 2.25)	0.656
	**Normal weight**	Ref.		Ref.	
**Hemorrhage/need for transfusion** ^f^					
	**Morbidly obese**	**1.78 (1.17, 2.73)**	**0.007**	**1.77 (1.12, 2.79)**	**0.014**
	**Obese**	1.19 (0.81, 1.75)	0.386	1.22 (0.81, 1.86)	0.341
	**Normal weight**	Ref.		Ref.	
**VTE** ^f^					
	**Morbidly obese**	2.00 (0.94, 4.25)	0.072	1.83 (0.84, 4.00)	0.130
	**Obese**	1.36 (0.64, 2.88)	0.419	1.44 (0.65, 3.17)	0.369
	**Normal weight**	Ref.		Ref.	
**AKI** ^f^					
	**Morbidly obese**	1.73 (0.98, 3.05)	0.058	1.65 (0.86, 3.15)	0.130
	**Obese**	1.24 (0.71, 2.18)	0.448	0.95 (0.50, 1.81)	0.864
	**Normal weight**	Ref.		Ref.	
**AMI** ^f^					
	**Morbidly obese**	NA		NA	
	**Obese**	1.35 (0.17, 10.67)	0.773	1.65 (0.22, 12.46)	0.625
	**Normal weight**	Ref.		Ref.	
**CVA** ^f^					
	**Morbidly obese**	1.47 (0.94, 2.30)	0.089	1.36 (0.82, 2.26)	0.235
	**Obese**	1.04 (0.69, 1.56)	0.841	1.11 (0.72, 1.70)	0.636
	**Normal weight**	Ref.		Ref.	
**ARDS/respiratory failure** ^f^					
	**Morbidly obese**	**1.72 (1.15, 2.56)**	**0.008**	1.50 (0.96, 2.34)	0.076
	**Obese**	0.99 (0.67, 1.45)	0.953	1.01 (0.67, 1.55)	0.946
	**Normal weight**	Ref.		Ref.	
**Mechanical ventilation ≥ 96 hours** ^f^					
	**Morbidly obese**	**2.33 (1.48, 3.66)**	**<0.001**	**2.14 (1.28, 3.58)**	**0.003**
	**Obese**	0.88 (0.53, 1.46)	0.615	0.84 (0.47, 1.48)	0.540
	**Normal weight**	Ref.		Ref.	

Abbreviations: LOS, length of hospital stay; SSI, surgical site infection; VTE, venous thromboembolism; AKI, acute kidney injury; AMI, acute myocardial infarction; CVA, cerebrovascular accident; ARDS, acute respiratory distress syndrome; CKD, chronic kidney disease; COPD, chronic obstruction pulmonary disease; ISS, injury severity score; NA, not applicable; ref, reference; aOR, adjusted odd ratio; CI, confidence interval.

P-values < 0.05 are shown in bold.

^a^ Excluding patients with in-hospital mortality.

^b^ Adjusted for age group, sex, insurance status, household income, smoking, diabetes, hypertension, CKD, ischemic heart disease, congestive heart failure, atrial fibrillation, anemia, COPD, cerebrovascular disease, peripheral vascular disease, severe liver disease, rheumatic disease, coagulopathy, any malignancy, fracture type, pneumothorax, ISS, hospital bed size, hospital location/teaching status and hospital region.

^c^ Adjusted for age group, sex, insurance status, household income, smoking, diabetes, hypertension, CKD, ischemic heart disease, congestive heart failure, atrial fibrillation, anemia, COPD, cerebrovascular disease, peripheral vascular disease, coagulopathy, any malignancy, fracture type, ISS, hospital location/teaching status and hospital region.

^d^ Adjusted for smoking, CKD, ischemic heart disease, congestive heart failure, atrial fibrillation, coagulopathy and fracture type.

^e^ Adjusted for household income, smoking, hypertension, atrial fibrillation, rheumatic disease, coagulopathy, fracture type, ISS, hospital location/teaching status and hospital region.

^f^ Adjusted for age group, household income, CKD, congestive heart failure, atrial fibrillation, COPD, cerebrovascular disease, coagulopathy, fracture type, ISS, hospital bed size, hospital location/teaching status and hospital region.

Characteristic

^g^ Morbidly obese is defined as BMI > 40 kg/m^2^, obese is defined as ≤ 30 BMI ≤ 40 kg/m^2^, and normal weight is 19 ≤ BMI <30 kg/m^2^.

### Associations between obesity status and adverse events stratified by fracture type

**Fig 2:**
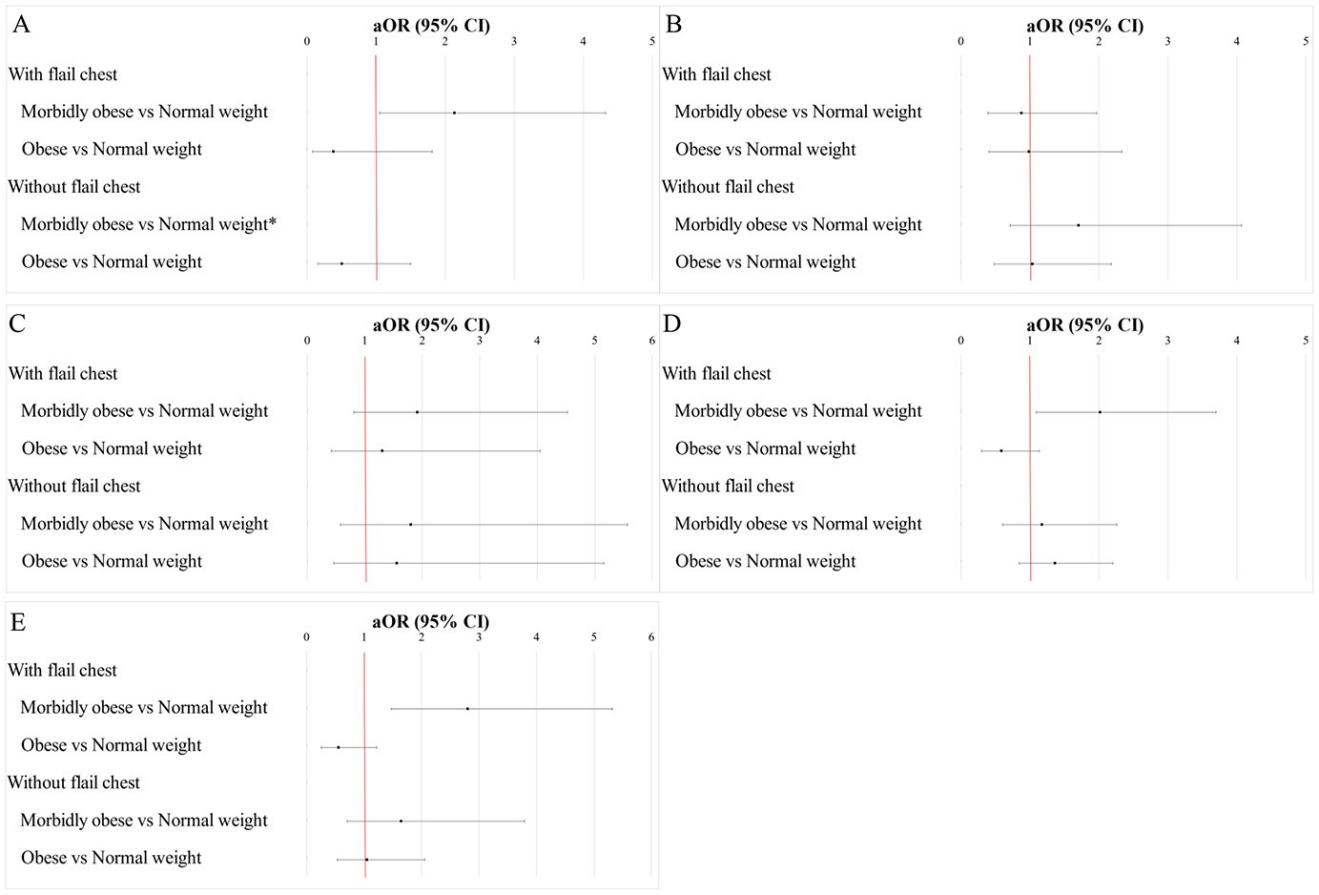
Stratified associations between obesity status and selected procedure and complications. (A) tracheostomy, (B) pneumonia, (C) VTE, (D) ARDS/respiratory failure, (E) mechanical ventilation ≥ 96 hours. The data were adjusted for age group, household income, smoking, CKD, congestive heart failure, atrial fibrillation, COPD, cerebrovascular disease, coagulopathy, hospital bed size, hospital location/teaching status, and hospital region. Abbreviation: ARDS, acute respiratory distress syndrome; VTE, venous thromboembolism; CKD, chronic kidney disease; COPD, chronic obstruction pulmonary disease; ref, reference; aOR, adjusted odd ratio; CI, confidence interval.

Fig 2A to 2E, along with S5 Tables in S1 File, show the associations between obesity status and adverse events. When adjusted possible confounders, compared to normal weight, morbid obesity was significantly associated with increased odds for tracheostomy (aOR, 2.13, 95% CI: 1.05, 4.32) (Fig 2A), ARDS/respiratory failure (aOR, 2.01, 95% CI: 1.09, 3.70) (Fig 2D) and mechanical ventilation ≥ 96 hours (aOR, 2.80, 95% CI: 1.47, 5.32) (Fig 2E) among patients with flail chest (all p < 0.05). In contrast, morbid obesity had no significant associations with the two adverse respiratory outcomes among those without a flail chest. In addition, morbid obesity was not significantly associated with pneumonia (Fig 2B) or VTE (Fig 2C) in patients both with and without flail chest (all p > 0.05).

## Discussion

In this investigation of patients with multiple rib fractures who underwent SSRF, we found that morbid obesity was independently associated with a list of adverse in-hospital outcomes. This included an increased LOS, higher total hospital costs, and increased risk of adverse events when compared to normal weight individuals. Among patients with flail chest, morbid obesity posed more than a two-fold risk for adverse respiratory outcomes, including tracheostomy, ARDS/respiratory failure, and prolonged mechanical ventilation. However, among patients without flail chest, no independent associations were found between morbidly obese status and respiratory outcomes.

The results of the present study align with previous orthopedic trauma research, indicating that obesity has an adverse impact on in-hospital outcomes [8, 15]. Specific to rib fractures, Lin et al. [10] conducted a study on thoracic trauma in Taiwan. Their findings revealed that the extent of rib fractures significantly correlates with prolonged LOS in both the ICU and hospital, irrespective of a patient’s weight/BMI. Another study reported that both ICU and hospital stays were significantly longer in orthopedic trauma patients who were obese. This was attributed not only to frequent intra- and post-operative complications but also to surgeons’ hesitancy in proceeding with surgical interventions, especially definitive fixation, in obese patients [8]. This delay and the ensuing complications increased LOS and escalated hospital costs.

However, a contrasting perspective study from a femur fracture fixation study where, despite the association of obesity with prolonged operative durations, postoperative complications between obese and non-obese patients did not differ significantly. This included repeat surgeries and surgeries for infection up to 90 days postoperatively [3]. Those authors postulated that patient positioning, intraoperative imaging, and difficulties in fracture reduction demanded added surgical effort and time, contributing directly to the longer duration of surgery. High-BMI surgical patients in a study of intertrochanteric hip fracture also had significantly longer operative durations [15].

On the other hand, in an interesting contradiction to the acknowledged association between hip fracture and high mortality, the “obesity paradox” shows that high BMI is positively associated with survival among older adults with chronic diseases. It suggests that a higher BMI, often linked to detrimental health effects, is correlated with improved survival rates among older adults with chronic illnesses. Notably, older adults who sustained hip fractures and were overweight or obese faced a lower risk of mortality compared to their underweight and malnutrition counterparts. This was evident even as their one-year survival rates improved and they were discharged to their homes, resuming independent living [16]. Since mortality rates are low in the setting of rib fracture surgeries, our present study examined only in-patient outcomes and did not address possible benefits associated with a higher BMI status. Meanwhile, other studies report that hip fracture surgeries in obese patients take significantly longer operative time and demand more work from surgeons compared to their non-obese counterparts [3], which ultimately leads to increased LOS and higher medical bills. In fact, among the various patient-related factors affecting surgical durations, a BMI exceeding 30 stands out as the most influential [4].

The present study did not assess operative times due to a lack of information. Longer operative times are associated with perioperative complications in all patients receiving operative procedures, and the risk is believed to be notably higher in obese individuals [17]. Further, in our analyses, morbid obesity and obesity were associated with longer hospital stays total costs, and respiratory complications in those with flail chest. Similarly, in a study of intertrochanteric hip fracture that reported higher operative times, more systemic complications, especially respiratory complications and wound infections that sometimes led to sepsis, occurred in obese and morbidly obese patients; those with BMI over 40 had a much higher rate of respiratory complications than those who were obese or non-obese [15]. A study of patients with femoral shaft fractures also compared normal-weight patients with overweight, obese, and morbidly obese patients, finding that systemic complications developed in 9% of normal patients and escalated to 23% of morbidly obese patients, who also had a 10% mortality rate (20% in the subset of polytraumatized patients) [18]. In that study, morbid obesity was particularly associated with increased odds of ARDS and sepsis. In a study of total knee arthroplasty, obesity was associated with higher rates of local complications such as prosthetic joint infection [2]. Higher rates of postoperative complications in obese patients are also shown for wound infection and deep vein thrombosis (DVT) [19–21]. Despite these reports of obesity-associated local and systemic complications of orthopedic surgeries, Ri et al. [17] concluded that obesity poses a greater risk of short-term complications but may not exert adverse effects on long-term surgical outcomes, which would depend on the type of surgery, expected surgical outcomes and extent of obesity, as well as patients’ overall health status. That said, obesity might not act alone and shall be evaluated along with other perioperative factors.

Traumatic rib fractures—the focus of the present study—are shown to be particularly severe in older adults who have experienced trauma, usually requiring ventilator support and intensive care [22]. Thoracic trauma is known to lead to respiratory failure, pneumonia, pleural sepsis, and death, and mortality will be even higher if multiple injuries are involved [23]. Multiple rib fractures are shown in a previous study to be associated with pulmonary complications, including lung contusions, pneumothorax, hemothorax, and pneumohemothorax [10]. Obesity is known to be associated with impaired pulmonary function [24]. Such respiratory issues begin with the accumulation of adipose tissue, decreased lung volume, and reduced effectiveness of the respiratory muscles, together leading to inspiratory overload, increasing oxygen consumption and respiratory effort as well as the expenditure of respiratory energy [24]. It is not surprising then that morbid obesity would further increase the risk of respiratory failure/ARDS and prolonged mechanical ventilation in the setting of SSRF for multiple rib fractures.

In this analysis, morbid obesity was significantly associated with increased odds for tracheostomy, ARDS/respiratory failure, and mechanical ventilation for about 96 hours only in patients with a flail chest. Patients with thoracic trauma that includes multiple rib fractures sometimes have a flail chest when a portion of the rib cage becomes detached from the chest wall. This may be accompanied by difficulty breathing, and internal bleeding and may also result in pneumothorax. A study of 407 patients with chest trauma involving multiple rib fractures with and without flail chest found that nearly 20% had flail chest and those patients more commonly had pneumothorax [25]. People who are morbidly obese often have more adipose tissue in the area of the chest wall. This extra fat might stiffen and make the chest wall less flexible, which further limits chest wall mobility during respiration. The combination of flail segments and reduced chest wall mobility brought on by morbid obesity, presumably, can exacerbate respiratory compromise and raise the risk of unfavorable consequences in patients with flail chest.

In our results, morbid obesity was not significantly associated with the risk of AKI, despite the well-established association between obesity and susceptibility to kidney disease [26]. Another study indicated that increased BMI was associated with a 20% risk of AKI in critically ill patients with ARDS [27]. Morbidly obese patients often have reduced lung capacity and increased oxygen demand, which can compromise respiratory function. This respiratory insufficiency can lead to suboptimal oxygenation and ventilation, resulting in tissue hypoxia. Such hypoxia can negatively impair kidney functionality, potentially leading to the onset of AKI [28].

### Strengths and limitations

The nationwide representation of our study stands as a significant strength. Nevertheless, the observational and retrospective nature of the study limits causal inferences and broad applicability. The proportion of obese patients in our study population appeared much lower than the known prevalence. This lower proportion of obese patients is likely attributable to the inherent limitations associated with administrative healthcare databases, such as potential undercoding or underdiagnosis of obesity-related conditions. Further, potential coding discrepancies arising from the ICD code system and the absence of detailed patient information, including intraoperative parameters and clinical laboratory data, restrict the interpretation. Additionally, the study lacks follow-up data for long-term post-discharge outcomes, necessitating caution while interpreting our findings.

## Conclusions

Among patients undergoing SSRF for multiple rib fractures, morbid obesity stands as an independent factor precipitating prolonged hospital stays and elevated hospital expenses compared to normal weight individuals. Furthermore, morbid obesity is also independently associated with adverse respiratory outcomes, including tracheostomy, ARDS/respiratory failure, and prolonged mechanical ventilation among patients with flail chest. These findings may help clinicians improve preoperative risk evaluations for patients with multiple rib fractures awaiting SSRF.

## Supporting information

S1 File (DOC)
