# Supplementary material for: Impact of obesity on outcomes after surgical stabilization of multiple rib fractures: Evidence from the US nationwide inpatient sample
Source: PLoS One. 2024 Feb 29;19(2):e0299256. doi: 10.1371/journal.pone.0299256 (PMC10903825; doi:10.1371/journal.pone.0299256)
Supplement: S1 File — (DOC) [file pone.0299256.s001.doc]

**Supplemental Table S1. ICD codes used in the study.**

|  | **ICD-9 code** | **ICD-10 code** |
| --- | --- | --- |
| Flail chest | **CM**: 807.4 | **CM**: S22.5 |
| Multiple rib fracture | **CM**: 807.02-807.09, 807.12-807.19 | **CM**: S22.4 |
| SSRF | **PCS:** 78.51, 79.19, 79.39 | **PCS:** 0PH104Z, 0PH134Z, 0PH144Z, 0PH204Z, 0PH234Z, 0PH244Z, 0PS104Z, 0PS134Z, 0PS144Z, 0PS204Z, 0PS234Z, 0PS244Z |
| Single rib fracture | **CM**: 807.01, 807.11 | **CM**: S22.3 |
| Brain or abdominal injury | **CM**: 781.4, 907.3, 873, 953.0 | **CM**: S01-S09, S14.0-S14.2, S34.0, S34.1 |
| Morbidly obese (>40 kg/m^2^) | **CM**: 278.01, V85.4 | **CM**: E66.01, E66.2, Z684 |
| Obese (30 ≤ BMI ≤40 kg/m^2^) | **CM**: 278.00, V85.3 | **CM**: E66.09, E66.1, E66.8, E66.9, Z68.3 |
| Normal weight (19 ≤ BMI <30 kg/m^2^) | **CM**: V85.1, V85.21–V85.25 | **CM**: Z68.2 |
| Underweight (<19kg/m^2^) | **CM**: V85.0 | **CM**: Z68.1 |
| Tracheostomy | **CM**: V44.0, V55.0,519.0, 519.00, 519.01, 519.02, 519.09 | **CM**: J95.0, Z93.0  **PCS:** 0B110F4, 0B110Z4, 0B113Z4, 0B114F4, 0B114Z4 |
| Pneumonia | **CM**: 073.0, 115.15, 115.95, 480, 481, 482, 483, 484.7, 484.8, 485, 486, 514, 517 | **CM**: J12–J18 |
| SSI | **CM**: 998.5 | **CM**: T81.4 |
| Sepsis | **CM**: 995.9, 996.64, 038, 999.3, 790.7, 041.x, 785.52 | **CM**: R78.81, A41, R65.2, T81.4, T80.2, A42.7, A22.7, B37.7, A26.7, A28.2, A54.86, B00.7, A32.7, A24.1, A39.2, A20.7, A21.7, A48.3 |
| Hemorrhage/need for transfusion | **CM**: 459.0, 285.1, 998.1  **PCS:** 39.98, 99.0 | **CM**: R58, D62  **PCS:** 0W380ZZ, 0W383ZZ, 0W384ZZ, 30233H, 30233J, 30233K, 30233L, 30233M, 30233N, 30233P, 30233R, 30233T, 30233V, 30233W |
| VTE | **CM**: 415, 451-453, 671, 673, 997.2 | **CM**: I260, I269, I801-803, I808, I809, I820-I823, I828, I829, O082, O223, O871, O882, I81, I82 |
| AKI | **CM**: 584  DXCCS: 157 | **CM**: N17  DXCCSR_GEN002 >0 |
| AMI | **CM**: 410  DXCCS: 100 | **CM**: I21  DXCCSR_CIR009 >0 |
| CVA | **CM**: 430, 431, 433.01, 433.10, 433.11, 433.21, 433.31, 433.81, 433.91, 434.00, 434.01, 434.11, 434.91, 436  DXCCS: 109 | **CM**: I60, I61, I63, I69  DXCCSR_CIR020 >0 |
| ARDS/respiratory failure | **CM**: 518.5, 518.81-518.84 | **CM**: J80, J81.0, J95.2-J95.8, J96.00, J96.90 |
| Mechanical ventilation ≥ 96 hours | **PCS**: 96.72 | **PCS**: 5A1955Z |
| Smoking | **CM**: V15.82, 292.0, 292.89, 292.9, 305.1, 989.84 | **CM**: Z71.6, Z72.0, Z86.4, Z87.891, F17, O99.33, T65.2 |
| Diabetes | CM_DM=1, CM_DMCX=1 | **CM**: E10-E14 |
| Hypertension | CM_HTN_C=1 | **CM**: I10 |
| CKD | **CM**: 403.01, 403.11, 403.91, 404.02, 404.03, 404.12, 404.13, 404.92, 404.93, 582, 583.0-583.7, 585, 586, 588.0, V42.0, V45.1, V56 | **CM**: I12.0, I13.1, N03.2-N03.7, N05.2-N05.7, N18, N19, N25.0, Z49.0-Z49.2, Z94.0, Z99.2 |
| Ischemic heart disease | **CM**: 410–414 | **CM**: I25 |
| Congestive heart failure | CM_CHF=1 | **CM**: I09.9, I11.0, I13.0, I13.2, I25.5, I42.0, I42.5-I42.9, I43, I50, P29.0 |
| Atrial fibrillation | **CM**: I48 | **CM**: 427.31 |
| Anemia | CM_ANEMDEF=1 | **CM**: D60, D61, D63, D64 |
| COPD | CM_CHRNLUNG=1 | **CM**: J40-44 |
| Cerebrovascular disease | **CM**: 362.34, 430-438 | **CM**: G45, G46, H34.0, I60-I69 |
| Peripheral vascular disease | **CM**: 093.0, 437.3, 440, 441, 443.1-443.9, 447.1, 557.1, 557.9, V43.4 | **CM**: I70, I71, I73.1, I73.8, I73.9, I77.1, I79.0, I79.2, K55.1, K55.8, K55.9, Z95.8, Z95.9 |
| Severe liver disease | **CM**: 456.0–456.2, 572.2–572.8 | **CM**: I85.0, I85.9, I86.4, I98.2, K70.4, K71.1, K72.1, K72.9, K76.5, K76.6, K76.7 |
| Rheumatic disease | **CM**: 446.5, 710.0-710.4, 714.0-714.2, 714.8, 725  CM_ARTH=1 | **CM**: M05, M06, M31.5, M32-M34, M35.1, M35.3, M36.0 |
| Coagulopathy | CM_COAG=1 | **CM**: D65-69 |
| Any malignancy | **CM**: 140-172, 174-195.8, 200-208, 238.6 | **CM**: C00-C26, C30-C34, C37-C41, C43, C45-C58, C60-C76, C81-C85, C88, C90-C97 |
| Pneumothorax | **CM**: 512 | **CM**: J93 |

Abbreviations: SSRF, surgical stabilization of rib fracture; SSI, surgical site infection; VTE, venous thromboembolism; AKI, acute kidney injury; AMI, acute myocardial infarction; CVA, cerebrovascular accident; ARDS, acute respiratory distress syndrome; CKD, chronic kidney disease; COPD, chronic obstructive pulmonary disease; ICD, International Classification of Diseases; CM, Clinical Modification; PCS, Procedure Coding System.

**Supplemental Table S2. Hospital bed size categories.**

| **Region** | **Location and teaching status** | **Hospital bed size** | | |
| --- | --- | --- | --- | --- |
|  |  | **Small** | **Medium** | **Large** |
| Northeast | Rural | 1–49 | 50–99 | 100+ |
|  | Urban, nonteaching | 1–124 | 125–199 | 200+ |
|  | Urban, teaching | 1–249 | 250–424 | 425+ |
| Midwest | Rural | 1–29 | 30–49 | 50+ |
|  | Urban, nonteaching | 1–74 | 75–174 | 175+ |
|  | Urban, teaching | 1–249 | 250–374 | 375+ |
| South | Rural | 1–39 | 40–74 | 75+ |
|  | Urban, nonteaching | 1–99 | 100–199 | 200+ |
|  | Urban, teaching | 1–249 | 250–449 | 450+ |
| West | Rural | 1–24 | 25–44 | 45+ |
|  | Urban, nonteaching | 1–99 | 100–174 | 175+ |
|  | Urban, teaching | 1–199 | 200–324 | 325 |

**Supplementary Table S3. Associations between obesity status and LOS, unfavorable discharge, in-hospital mortality, and hospital costs.**

| **Variables** | **LOS, days ^a^** | | **Unfavorable discharge ^a^** | | **In-hospital mortality** | | **Hospital cost** | |
| --- | --- | --- | --- | --- | --- | --- | --- | --- |
|  | **Univariate** | **Multivariate** | **Univariate** | **Multivariate** | **Univariate** | **Multivariate** | **Univariate** | **Multivariate** |
|  | **Beta (95% CI)** | **aBeta (95% CI)** | **OR (95% CI)** | **aOR (95% CI)** | **OR (95% CI)** | **aOR (95% CI)** | **Beta (95% CI)** | **aBeta (95% CI)** |
| **Morbidly obese**  **(>40 kg/m^2^)** | **0.07 (0.07, 0.07)** | **0.07 (0.06, 0.07)** | 1.39 (0.91, 2.11) | 1.43 (0.86, 2.36) | 1.22 (0.16, 9.31) | 0.79 (0.16, 3.82) | **46.85 (7.26, 86.44)** | **47.35 (38.55, 56.14)** |
| **Obese**  **(30 ≤ BMI ≤40 kg/m^2^)** | **0.02 (0.00, 0.03)** | -0.02 (-0.03, 0.00) | 1.08 (0.72, 1.62) | 0.90 (0.56, 1.43) | NA | NA | -17.72 (-42.76, 7.32) | **-7.22 (-12.25, -2.19)** |
| **Normal weight**  **(19 ≤ BMI <30 kg/m^2^)** | Ref. | Ref. | Ref. | Ref. | Ref. | Ref. | Ref. | Ref. |
| **Age, years** |  |  |  |  |  |  |  |  |
| 20-39 | Ref. | Ref. | Ref. | Ref. | Ref. |  | Ref. |  |
| 40-59 | **0.04 (0.04, 0.04)** | **0.04 (0.04, 0.05)** | 1.32 (0.91, 1.93) | 1.36 (0.90, 2.05) | NA |  | 6.78 (-15.90, 29.46) |  |
| 60+ | **0.30 (0.30, 0.30)** | **0.24 (0.23, 0.24)** | **4.45 (3.08, 6.42)** | **3.58 (2.36, 5.44)** | NA |  | **27.46 (0.51, 54.41)** |  |
| **Sex** |  |  |  |  |  |  |  |  |
| Male | **-0.08 (-0.09, -0.08)** | **-0.07 (-0.07, -0.07)** | **0.68 (0.55, 0.84)** | **0.70 (0.55, 0.89)** | 0.49 (0.20, 1.22) |  | 14.02 (-3.90, 31.95) |  |
| Female | Ref. | Ref. | Ref. | Ref. | Ref. |  | Ref. |  |
| **Insurance status** |  |  |  |  |  |  |  |  |
| Medicare/Medicaid | Ref. | Ref. | Ref. | Ref. | Ref. |  | Ref. |  |
| Private including HMO | **-0.15 (-0.15, -0.14)** | **-0.05 (-0.05, -0.05)** | **0.52 (0.42, 0.63)** | **0.76 (0.59, 0.96)** | 0.59 (0.22, 1.56) |  | 2.90 (-5.32, 11.12) |  |
| Self-pay/no-charge/other | **-0.21 (-0.21, -0.21)** | **-0.08 (-0.08, -0.08)** | **0.37 (0.28, 0.49)** | **0.64 (0.45, 0.91)** | 0.50 (0.16, 1.57) |  | **15.47 (2.70, 28.25)** |  |
| **Household income** |  |  |  |  |  |  |  |  |
| Quartile1 | **0.08 (0.08, 0.08)** | **0.10 (0.10, 0.10)** | **1.48 (1.10, 1.98)** | **1.80 (1.28, 2.53)** | 0.73 (0.19, 2.76) |  | **17.04 (12.33, 21.75)** | **26.95 (21.89, 32.01)** |
| Quartile2 | **0.09 (0.08, 0.09)** | **0.09 (0.09, 0.09)** | **1.53 (1.15, 2.04)** | **1.70 (1.21, 2.37)** | 0.46 (0.10, 2.19) |  | **-8.07 (-13.07, -3.06)** | -4.30 (-9.69, 1.09) |
| Quartile3 | **0.08 (0.08, 0.09)** | **0.08 (0.08, 0.09)** | **1.52 (1.15, 2.00)** | **1.62 (1.19, 2.21)** | 1.64 (0.61, 4.38) |  | -7.09 (-14.40, 0.23) | -1.18 (-8.57, 6.21) |
| Quartile4 | Ref. | Ref. | Ref. | Ref. | Ref. |  | Ref. | Ref. |
| **Smoking** |  |  |  |  |  |  |  |  |
| No | Ref. | Ref. | Ref. | Ref. | Ref. | Ref. | Ref. | Ref. |
| Yes | **-0.06 (-0.06, -0.06)** | **-0.05 (-0.05, -0.05)** | **0.74 (0.61, 0.91)** | **0.77 (0.61, 0.97)** | **0.29 (0.10, 0.90)** | **0.30 (0.10, 0.91)** | **-33.35 (-50.08, -16.63)** | **-20.59 (-23.76, -17.42)** |
| **Major comorbidities** |  |  |  |  |  |  |  |  |
| Diabetes | **0.11 (0.11, 0.12)** | **0.04 (0.04, 0.04)** | **1.67 (1.32, 2.11)** | 1.27 (0.95, 1.68) | 0.36 (0.05, 2.70) |  | 15.62 (-7.61, 38.86) |  |
| Hypertension | **0.08 (0.08, 0.08)** | **-0.01 (-0.01, 0.00)** | **1.46 (1.19, 1.78)** | 0.94 (0.74, 1.20) | 0.55 (0.18, 1.67) |  | **-17.62 (-34.42, -0.81)** | **-14.65 (-19.14, -10.15)** |
| CKD | **0.32 (0.30, 0.33)** | **0.14 (0.14, 0.15)** | **3.74 (2.30, 6.09)** | **1.86 (1.05, 3.29)** | **8.97 (2.96, 27.17)** | **3.71 (1.13, 12.12)** | 35.12 (-10.32, 80.55) |  |
| Ischemic heart disease | **0.14 (0.14, 0.15)** | **-0.01 (-0.01, -0.01)** | **1.86 (1.38, 2.50)** | 0.98 (0.68, 1.42) | **7.52 (3.00, 18.83)** | **4.84 (1.54, 15.27)** | -12.30 (-37.23, 12.62) |  |
| Congestive heart failure | **0.29 (0.29, 0.29)** | **0.12 (0.12, 0.12)** | **3.33 (2.11, 5.25)** | 1.69 (0.99, 2.89) | **5.43 (1.53, 19.31)** | 1.40 (0.34, 5.73) | 25.43 (-12.47, 63.32) |  |
| Atrial fibrillation | **0.24 (0.24, 0.25)** | **0.08 (0.08, 0.08)** | **2.81 (2.04, 3.87)** | 1.39 (0.94, 2.04) | **5.02 (1.93, 13.07)** | 2.13 (0.64, 7.13) | **62.03 (28.11, 95.95)** | **54.48 (48.61, 60.34)** |
| Anemia | **0.18 (0.18, 0.18)** | **0.04 (0.04, 0.05)** | **2.14 (1.29, 3.55)** | 1.20 (0.68, 2.12) | 2.19 (0.30, 16.03) |  | 45.34 (-1.40, 92.08) |  |
| COPD | **0.10 (0.10, 0.11)** | **0.07 (0.06, 0.07)** | **1.57 (1.14, 2.17)** | 1.41 (0.99, 2.02) | 1.40 (0.46, 4.25) |  | 16.24 (-14.29, 46.76) |  |
| Cerebrovascular disease | **0.21 (0.21, 0.21)** | **0.12 (0.12, 0.12)** | **2.44 (1.29, 4.61)** | 1.81 (0.80, 4.09) | 4.01 (0.52, 30.80) |  | 37.34 (-36.54, 111.22) |  |
| Peripheral vascular  disease | **0.18 (0.18, 0.18)** | **0.07 (0.07, 0.08)** | **2.12 (1.27, 3.54)** | 1.45 (0.78, 2.72) | 2.37 (0.30, 18.83) |  | 17.40 (-22.74, 57.54) |  |
| Severe Liver disease | **0.08 (0.08, 0.08)** | **0.22 (0.22, 0.23)** | 1.43 (0.41, 4.92) |  | NA |  | 151.01 (-111.47, 413.48) |  |
| Rheumatic disease | **0.14 (0.14, 0.14)** | **0.02 (0.02, 0.02)** | 1.80 (0.87, 3.71) |  | 4.33 (0.55, 34.15) |  | **-42.68 (-73.09, -12.28)** | **-27.23 (-39.85, -14.61)** |
| Coagulopathy | **0.15 (0.14, 0.15)** | **0.05 (0.05, 0.05)** | **1.87 (1.07, 3.28)** | 1.27 (0.66, 2.44) | **6.14 (1.37, 27.44)** | 2.10 (0.36, 12.15) | **103.96 (28.38, 179.54)** | **82.68 (81.07, 84.28)** |
| Any malignancy | **0.30 (0.29, 0.30)** | **0.19 (0.19, 0.19)** | **3.43 (1.47, 8.01)** | **2.51 (1.07, 5.90)** | NA |  | -6.93 (-69.07, 55.22) |  |
| **Fracture type** |  |  |  |  |  |  |  |  |
| With flail chest | **0.11 (0.10, 0.11)** | **0.08 (0.08, 0.09)** | **1.63 (1.35, 1.98)** | **1.58 (1.26, 1.97)** | **3.02 (1.24, 7.36)** | 2.57 (0.98, 6.70) | **88.99 (66.30, 111.68)** | **79.37 (74.50, 84.24)** |
| Without flail chest | Ref. | Ref. | Ref. | Ref. | Ref. | Ref. | Ref. | Ref. |
| **Pneumothorax** |  |  |  |  |  |  |  |  |
| No | Ref. | Ref. | Ref. |  | Ref. |  | Ref. |  |
| Yes | **0.07 (0.07, 0.07)** | **0.08 (0.08, 0.08)** | 1.35 (0.90, 2.02) |  | 2.65 (0.86, 8.15) |  | -4.15 (-43.58, 35.27) |  |
| **ISS** |  |  |  |  |  |  |  |  |
| <9 | Ref. | Ref. | Ref. | Ref. | Ref. |  | Ref. | Ref. |
| ≥9 | **0.19 (0.19, 0.20)** | **0.15 (0.14, 0.15)** | **3.28 (1.95, 5.51)** | **2.79 (1.64, 4.76)** | NA |  | **107.42 (89.42, 125.41)** | **68.50 (64.80, 72.20)** |
| **Hospital bed size** |  |  |  |  |  |  |  |  |
| Small | **-0.02 (-0.04, 0.00)** | **-0.07 (-0.09, -0.06)** | 0.91 (0.68, 1.21) |  | NA |  | 30.85 (-35.19, 96.89) |  |
| Medium | **-0.01 (-0.02, 0.00)** | **-0.02 (-0.03, -0.01)** | 0.95 (0.76, 1.20) |  | 0.59 (0.13, 2.55) |  | -5.57 (-29.29, 18.15) |  |
| Large | Ref. | Ref. | Ref. |  | Ref. |  | Ref. |  |
| **Hospital location/teaching status** |  |  |  |  |  |  |  |  |
| Rural | **-0.25 (-0.25, -0.25)** | **-0.22 (-0.22, -0.22)** | **0.19 (0.09, 0.39)** | **0.19 (0.08, 0.44)** | 3.31 (0.41, 26.81) |  | **-93.92 (-120.50, -67.35)** | **-74.85 (-77.43, -72.28)** |
| Urban nonteaching | **-0.10 (-0.11, -0.09)** | **-0.08 (-0.09, -0.07)** | **0.60 (0.45, 0.79)** | **0.67 (0.49, 0.90)** | 1.08 (0.46, 2.54) |  | 1.51 (-27.21, 30.22) | **4.29 (1.81, 6.76)** |
| Urban teaching | Ref. | Ref. | Ref. | Ref. | Ref. |  | Ref. | Ref. |
| **Hospital region** |  |  |  |  |  |  |  |  |
| Northeast | Ref. | Ref. | Ref. | Ref. | Ref. |  | Ref. | Ref. |
| South | **-0.06 (-0.07, -0.05)** | **-0.05 (-0.06, -0.04)** | 0.78 (0.58, 1.06) | 0.77 (0.53, 1.11) | 0.83 (0.31, 2.23) |  | -12.57 (-37.33, 12.19) | **-16.94 (-30.12, -3.75)** |
| Midwest | **-0.09 (-0.10, -0.08)** | **-0.08 (-0.09, -0.07)** | **0.65 (0.48, 0.88)** | **0.63 (0.44, 0.91)** | **0.20 (0.04, 0.96)** |  | 24.64 (-2.70, 51.97) | **21.99 (8.83, 35.15)** |
| West | **-0.10 (-0.11, -0.09)** | **-0.08 (-0.09, -0.07)** | **0.64 (0.48, 0.85)** | **0.65 (0.46, 0.91)** | **0.20 (0.04, 0.96)** |  | **42.06 (7.23, 76.89)** | **36.42 (23.24, 49.60)** |

Abbreviations: HMO, health maintenance organization; LOS, length of stay; CKD, chronic kidney disease; COPD, chronic obstructive pulmonary disease; ISS, injury severity score; NA, not applicable; Ref, reference; aOR, adjusted odd ratio; CI, confidence interval.

Significant values are shown in bold.

Variables that were significant in univariate regression were adjusted in multivariable models.

^a^ Excluding patients with in-hospital death.

**Supplementary Table S4. Associations between obesity status and adverse events.**

| **Variable** | **Adverse events, any** | | **Tracheostomy** | | **Pneumonia** | | **SSI** | |
| --- | --- | --- | --- | --- | --- | --- | --- | --- |
|  | **Univariate** | **Multivariate** | **Univariate** | **Multivariate** | **Univariate** | **Multivariate** | **Univariate** | **Multivariate** |
|  | **OR (95% CI)** | **aOR (95% CI)** | **OR (95% CI)** | **aOR (95% CI)** | **OR (95% CI)** | **aOR (95% CI)** | **OR (95% CI)** | **aOR (95% CI)** |
| **Morbidly obese**  **(>40 kg/m^2^)** | **1.69 (1.14, 2.52)** | **1.63 (1.02, 2.61)** | 1.60 (0.86, 2.98) | 1.32 (0.69, 2.55) | 1.41 (0.82, 2.44) | 1.15 (0.60, 2.20) | 1.89 (0.43, 8.25) | 2.19 (0.39, 12.19) |
| **Obese**  **(30 ≤ BMI ≤40 kg/m^2^)** | 1.17 (0.81, 1.69) | 1.13 (0.75, 1.69) | 0.51 (0.23, 1.16) | 0.44 (0.17, 1.11) | 1.10 (0.66, 1.83) | 1.04 (0.60, 1.81) | NA | NA |
| **Normal weight**  **(19 ≤ BMI <30 kg/m^2^)** | Ref. | Ref. | Ref. | Ref. | Ref. | Ref. | Ref. | Ref. |
| **Age, years** |  |  |  |  |  |  |  |  |
| 20-39 | Ref. | Ref. | Ref. | Ref. | Ref. | Ref. | Ref. | Ref. |
| 40-59 | 1.19 (0.91, 1.56) | 1.21 (0.91, 1.60) | 1.67 (0.92, 3.03) | 1.76 (0.97, 3.20) | 1.15 (0.79, 1.67) | 1.07 (0.72, 1.57) | **3.23 (1.66, 6.28)** | **2.91 (1.41, 6.00)** |
| 60+ | **1.56 (1.20, 2.04)** | **1.36 (1.02, 1.81)** | **2.22 (1.21, 4.10)** | **2.28 (1.19, 4.39)** | 1.28 (0.86, 1.91) | 1.09 (0.72, 1.65) | **4.32 (2.48, 7.55)** | **2.75 (1.37, 5.53)** |
| **Sex** |  |  |  |  |  |  |  |  |
| Male | 0.91 (0.76, 1.10) |  | **1.66 (1.10, 2.51)** |  | **1.33 (1.00, 1.78)** |  | 1.26 (0.47, 3.38) |  |
| Female | Ref. |  | Ref. |  | Ref. |  | Ref. |  |
| **Insurance status** |  |  |  |  |  |  |  |  |
| Medicare/Medicaid | Ref. |  | Ref. |  | Ref. |  | Ref. |  |
| Private including HMO | 0.99 (0.81, 1.20) |  | 1.12 (0.76, 1.65) |  | 0.97 (0.72, 1.29) |  | 0.98 (0.37, 2.60) |  |
| Self-pay/no-charge/other | 0.83 (0.65, 1.06) |  | 1.13 (0.72, 1.78) |  | 1.12 (0.78, 1.60) |  | 1.71 (0.59, 4.98) |  |
| **Household income** |  |  |  |  |  |  |  |  |
| Quartile1 | **1.88 (1.45, 2.45)** | **1.69 (1.25, 2.28)** | 1.59 (0.99, 2.55) | 1.65 (0.96, 2.85) | **1.73 (1.18, 2.52)** | **1.64 (1.09, 2.48)** | 0.13 (0.02, 1.11) | 0.11 (0.01, 1.01) |
| Quartile2 | **1.58 (1.24, 2.02)** | **1.48 (1.13, 1.96)** | 1.35 (0.85, 2.15) | 1.27 (0.75, 2.14) | 1.39 (0.95, 2.03) | 1.31 (0.89, 1.94) | 0.87 (0.31, 2.42) | 0.86 (0.29, 2.60) |
| Quartile3 | **1.84 (1.43, 2.36)** | **1.78 (1.35, 2.35)** | 1.18 (0.74, 1.90) | 1.10 (0.66, 1.83) | **1.43 (1.00, 2.05)** | 1.36 (0.93, 1.98) | 0.72 (0.25, 2.09) | 0.71 (0.23, 2.22) |
| Quartile4 | Ref. | Ref. | Ref. | Ref. | Ref. | Ref. | Ref. | Ref. |
| **Smoking** |  |  |  |  |  |  |  |  |
| No | Ref. |  | Ref. |  | Ref. |  | Ref. |  |
| Yes | 0.86 (0.72, 1.02) |  | **0.35 (0.23, 0.53)** |  | 0.90 (0.70, 1.16) |  | 0.57 (0.24, 1.33) |  |
| **Major comorbidities** |  |  |  |  |  |  |  |  |
| Diabetes | 1.25 (0.99, 1.58) |  | 1.02 (0.68, 1.55) |  | 0.79 (0.55, 1.13) |  | 0.50 (0.11, 2.28) |  |
| Hypertension | 1.06 (0.88, 1.26) |  | 0.77 (0.55, 1.08) |  | 0.91 (0.71, 1.17) |  | 1.92 (0.88, 4.18) |  |
| CKD | **2.50 (1.46, 4.30)** | 1.62 (0.85, 3.07) | 1.23 (0.57, 2.65) | 0.73 (0.28, 1.88) | 1.04 (0.53, 2.05) | 0.75 (0.37, 1.52) | 1.21 (0.16, 9.08) | 0.61 (0.07, 5.33) |
| Ischemic heart disease | 1.34 (0.99, 1.82) |  | 0.77 (0.47, 1.28) |  | 0.90 (0.59, 1.38) |  | 0.91 (0.21, 3.92) |  |
| Congestive heart failure | **2.17 (1.34, 3.53)** | 1.30 (0.76, 2.24) | 1.10 (0.49, 2.46) | 0.73 (0.29, 1.83) | 1.53 (0.86, 2.72) | 1.19 (0.61, 2.35) | 1.08 (0.14, 8.12) | 0.91 (0.08, 10.91) |
| Atrial fibrillation | **2.61 (1.83, 3.72)** | **1.99 (1.30, 3.07)** | **2.00 (1.26, 3.16)** | **1.70 (1.00, 2.88)** | **1.99 (1.35, 2.93)** | **1.67 (1.07, 2.62)** | **3.32 (1.23, 8.97)** | 2.10 (0.50, 8.75) |
| Anemia | 1.32 (0.79, 2.21) |  | 0.64 (0.20, 2.07) |  | **2.24 (1.21, 4.15)** |  | NA |  |
| COPD | **2.14 (1.57, 2.92)** | **2.12 (1.53, 2.95)** | **1.95 (1.25, 3.06)** | **1.87 (1.14, 3.06)** | **2.32 (1.62, 3.34)** | **2.35 (1.59, 3.49)** | 0.40 (0.05, 3.23) | 0.44 (0.06, 3.21) |
| Cerebrovascular disease | **3.07 (1.38, 6.85)** | **2.91 (1.26, 6.71)** | 1.68 (0.58, 4.90) | 1.48 (0.44, 4.95) | 1.83 (0.85, 3.92) | 1.63 (0.73, 3.63) | **25.33 (9.25, 69.33)** | **32.58 (8.54, 124.28)** |
| Peripheral vascular  disease | 1.15 (0.68, 1.92) |  | 0.72 (0.27, 1.95) |  | 0.40 (0.15, 1.08) |  | 1.69 (0.22, 12.71) |  |
| Severe liver disease | 0.93 (0.28, 3.10) |  | **3.63 (1.04, 12.58)** |  | 2.02 (0.59, 6.91) |  | **29.02 (5.96, 141.23)** |  |
| Rheumatic disease | 0.90 (0.45, 1.83) |  | 0.35 (0.05, 2.61) |  | 0.73 (0.22, 2.43) |  | 2.67 (0.32, 22.08) |  |
| Coagulopathy | **3.05 (1.63, 5.73)** | **2.78 (1.32, 5.88)** | 0.53 (0.19, 1.53) | 0.46 (0.15, 1.44) | **2.19 (1.12, 4.30)** | **2.10 (1.03, 4.27)** | 1.99 (0.29, 13.65) | 1.15 (0.09, 14.18) |
| Any malignancy | 1.14 (0.50, 2.58) |  | 0.56 (0.08, 4.15) |  | 2.03 (0.79, 5.22) |  | 4.31 (0.56, 33.16) |  |
| **Fracture type** |  |  |  |  |  |  |  |  |
| With flail chest | **2.64 (2.19, 3.18)** | **2.47 (2.02, 3.01)** | **3.17 (2.31, 4.34)** | **3.05 (2.22, 4.21)** | **2.12 (1.64, 2.73)** | **1.92 (1.47, 2.50)** | 1.21 (0.53, 2.72) | 1.06 (0.41, 2.74) |
| Without flail chest | Ref. | Ref. | Ref. | Ref. | Ref. | Ref. | Ref. | Ref. |
| **Pneumothorax** |  |  |  |  |  |  |  |  |
| No | Ref. |  | Ref. |  | Ref. |  | Ref. |  |
| Yes | 0.99 (0.65, 1.51) |  | 1.29 (0.69, 2.41) |  | 1.24 (0.73, 2.11) |  | 1.85 (0.43, 8.01) |  |
| **ISS** |  |  |  |  |  |  |  |  |
| <9 | Ref. | Ref. | Ref. | Ref. | Ref. | Ref. | Ref. | Ref. |
| ≥9 | **4.57 (2.90, 7.21)** | **3.64 (2.21, 6.00)** | **10.09 (1.40, 72.85)** | 6.58 (0.89, 48.87) | **18.15 (2.51, 131.40)** | **13.42 (1.81, 99.41)** | 1.31 (0.18, 9.71) | 0.99 (0.12, 8.11) |
| **Hospital bed size** |  |  |  |  |  |  |  |  |
| Small | **0.68 (0.52, 0.91)** | **0.60 (0.43, 0.82)** | 0.42 (0.17, 1.02) | 0.42 (0.17, 1.06) | 0.70 (0.44, 1.12) | 0.67 (0.39, 1.15) | **0.62 (0.39, 0.97)** | 0.75 (0.44, 1.31) |
| Medium | **0.70 (0.57, 0.87)** | **0.67 (0.54, 0.85)** | **0.62 (0.38, 0.99)** | 0.62 (0.38, 1.02) | 0.80 (0.55, 1.15) | 0.87 (0.61, 1.26) | 0.63 (0.26, 1.50) | 0.74 (0.25, 2.20) |
| Large | Ref. | Ref. | Ref. | Ref. | Ref. | Ref. | Ref. | Ref. |
| **Hospital location/teaching status** | |  |  |  |  |  |  |  |
| Rural | **0.28 (0.21, 0.39)** | **0.23 (0.16, 0.34)** | 0.63 (0.22, 1.80) | 0.75 (0.26, 2.12) | 1.01 (0.52, 1.96) | 1.18 (0.59, 2.40) | NA | NA |
| Urban nonteaching | **0.67 (0.55, 0.84)** | **0.63 (0.50, 0.79)** | 0.91 (0.60, 1.36) | 0.97 (0.65, 1.46) | 1.16 (0.85, 1.59) | 1.07 (0.78, 1.47) | 1.01 (0.35, 2.94) | 0.82 (0.25, 2.71) |
| Urban teaching | Ref. | Ref. | Ref. | Ref. | Ref. | Ref. | Ref. | Ref. |
| **Hospital region** |  |  |  |  |  |  |  |  |
| Northeast | Ref. | Ref. | Ref. | Ref. | Ref. | Ref. | Ref. | Ref. |
| South | 1.16 (0.86, 1.57) | 1.07 (0.75, 1.52) | 0.95 (0.55, 1.66) | 0.84 (0.47, 1.47) | 1.22 (0.77, 1.95) | 1.09 (0.66, 1.79) | 3.45 (0.44, 27.23) | 4.55 (0.34, 60.05) |
| Midwest | 1.23 (0.93, 1.63) | 1.23 (0.88, 1.72) | 0.82 (0.47, 1.42) | 0.81 (0.46, 1.44) | 1.13 (0.73, 1.73) | 1.09 (0.69, 1.74) | 3.62 (0.44, 29.57) | 6.57 (0.49, 88.82) |
| West | 0.87 (0.65, 1.16) | 0.85 (0.61, 1.21) | 0.82 (0.46, 1.44) | 0.83 (0.47, 1.47) | 1.00 (0.68, 1.48) | 1.07 (0.67, 1.72) | 4.06 (0.50, 32.93) | 5.84 (0.45, 76.37) |

Abbreviations: SSI, surgical site infection; HMO, health maintenance organization; CKD, chronic kidney disease; COPD, chronic obstructive pulmonary disease; ISS, injury severity score; NA, not applicable; Ref, reference; aOR, adjusted odd ratio; CI, confidence interval.

Significant values are shown in bold.

Variables that were significant in univariate regression were adjusted in multivariable models.

**Supplementary Table S4. Associations between obesity status and adverse events. (cont.)**

| **Variables** | **Sepsis** | | **Hemorrhage/need for transfusion** | | **VTE** | | **AKI** | |
| --- | --- | --- | --- | --- | --- | --- | --- | --- |
|  | **Univariate** | **Multivariate** | **Univariate** | **Multivariate** | **Univariate** | **Multivariate** | **Univariate** | **Multivariate** |
|  | **OR (95% CI)** | **aOR (95% CI)** | **OR (95% CI)** | **aOR (95% CI)** | **OR (95% CI)** | **aOR (95% CI)** | **OR (95% CI)** | **aOR (95% CI)** |
| **Morbidly obese**  **(>40 kg/m^2^)** | 1.56 (0.79, 3.06) | 1.39 (0.63, 3.06) | **1.78 (1.17, 2.73)** | **1.77 (1.12, 2.79)** | 2.00 (0.94, 4.25) | 1.83 (0.84, 4.00) | 1.73 (0.98, 3.05) | 1.65 (0.86, 3.15) |
| **Obese**  **(30 ≤ BMI ≤40 kg/m^2^)** | 1.10 (0.60, 2.03) | 1.16 (0.60, 2.25) | 1.19 (0.81, 1.75) | 1.22 (0.81, 1.86) | 1.36 (0.64, 2.88) | 1.44 (0.65, 3.17) | 1.24 (0.71, 2.18) | 0.95 (0.50, 1.81) |
| **Normal weight**  **(19 ≤ BMI <30 kg/m^2^)** | Ref. | Ref. | Ref. | Ref. | Ref. | Ref. | Ref. | Ref. |
| **Age** |  |  |  |  |  |  |  |  |
| 20-39 | Ref. | Ref. | Ref. | Ref. | Ref. | Ref. | Ref. | Ref. |
| 40-59 | 0.89 (0.51, 1.54) | 0.82 (0.47, 1.43) | 1.01 (0.73, 1.40) | 1.03 (0.74, 1.42) | 1.56 (0.69, 3.52) | 1.58 (0.68, 3.64) | **3.71 (1.81, 7.64)** | **3.72 (1.85, 7.49)** |
| 60+ | 1.19 (0.69, 2.05) | 0.91 (0.50, 1.65) | 1.31 (0.95, 1.79) | 1.24 (0.89, 1.72) | **2.35 (1.04, 5.31)** | 2.23 (0.97, 5.15) | **8.39 (4.09, 17.23)** | **5.96 (2.91, 12.20)** |
| **Sex** |  |  |  |  |  |  |  |  |
| Male | 1.00 (0.69, 1.45) |  | 0.87 (0.71, 1.07) |  | **2.19 (1.18, 4.06)** |  | **2.07 (1.40, 3.08)** |  |
| Female | Ref. |  | Ref. |  | Ref. |  | Ref. |  |
| **Insurance status** |  |  |  |  |  |  |  |  |
| Medicare/Medicaid |  |  |  |  |  |  |  |  |
| Private including HMO | 0.71 (0.48, 1.04) |  | 1.01 (0.81, 1.24) |  | 1.51 (0.92, 2.48) |  | 0.84 (0.62, 1.15) |  |
| Self-pay/no-charge/other | 1.01 (0.64, 1.59) |  | 0.91 (0.69, 1.21) |  | 0.96 (0.49, 1.88) |  | 0.70 (0.45, 1.08) |  |
| **Household income** |  |  |  |  |  |  |  |  |
| Quartile1 | **2.02 (1.20, 3.41)** | **1.81 (1.04, 3.16)** | **1.69 (1.26, 2.28)** | **1.63 (1.18, 2.26)** | 0.88 (0.46, 1.69) | 0.74 (0.39, 1.43) | 1.42 (0.91, 2.20) | 1.66 (1.00, 2.77) |
| Quartile2 | 1.38 (0.79, 2.42) | 1.29 (0.71, 2.34) | **1.45 (1.08, 1.95)** | **1.40 (1.02, 1.91)** | 0.99 (0.54, 1.81) | 0.81 (0.43, 1.53) | 1.42 (0.92, 2.17) | 1.49 (0.92, 2.39) |
| Quartile3 | **2.20 (1.32, 3.67)** | **2.15 (1.26, 3.67)** | **1.59 (1.19, 2.13)** | **1.57 (1.15, 2.15)** | 1.12 (0.64, 1.97) | 0.98 (0.56, 1.70) | **1.70 (1.11, 2.62)** | **1.92 (1.20, 3.08)** |
| Quartile4 | Ref. | Ref. | Ref. | Ref. | Ref. | Ref. | Ref. | Ref. |
| **Smoking** |  |  |  |  |  |  |  |  |
| No | Ref. |  | Ref. |  | Ref. |  | Ref. |  |
| Yes | 0.73 (0.51, 1.04) |  | 0.82 (0.67, 1.01) |  | 0.63 (0.40, 1.01) |  | **0.65 (0.47, 0.91)** |  |
| **Major comorbidities** |  |  |  |  |  |  |  |  |
| Diabetes | 0.98 (0.63, 1.52) |  | 1.29 (0.99, 1.67) |  | 0.84 (0.48, 1.46) |  | **2.18 (1.57, 3.03)** |  |
| Hypertension | 1.05 (0.75, 1.45) |  | 0.88 (0.73, 1.07) |  | 0.68 (0.45, 1.05) |  | **1.62 (1.21, 2.16)** |  |
| CKD | 1.82 (0.94, 3.52) | 1.13 (0.52, 2.44) | **1.72 (1.06, 2.80)** | 1.26 (0.71, 2.26) | 1.38 (0.55, 3.43) | 0.93 (0.35, 2.46) | **11.01 (6.89, 17.59)** | **6.94 (3.96, 12.14)** |
| Ischemic heart disease | 1.25 (0.75, 2.10) |  | 1.01 (0.72, 1.40) |  | 0.89 (0.43, 1.83) |  | **2.74 (1.88, 3.98)** |  |
| Congestive heart failure | **3.95 (2.28, 6.86)** | **2.80 (1.51, 5.20)** | 1.44 (0.90, 2.29) | 0.89 (0.52, 1.53) | 1.94 (0.84, 4.52) | 1.37 (0.55, 3.41) | **4.83 (3.04, 7.66)** | **2.27 (1.28, 4.02)** |
| Atrial fibrillation | **2.30 (1.44, 3.66)** | **1.79 (1.06, 3.02)** | **1.87 (1.39, 2.52)** | **1.45 (1.01, 2.09)** | 1.26 (0.61, 2.59) | 0.84 (0.40, 1.76) | **3.50 (2.35, 5.20)** | **2.04 (1.23, 3.37)** |
| Anemia | 1.19 (0.47, 3.03) |  | 0.62 (0.31, 1.21) |  | 1.30 (0.48, 3.50) |  | 1.32 (0.66, 2.61) |  |
| COPD | 1.41 (0.84, 2.36) | 1.43 (0.83, 2.47) | 1.19 (0.85, 1.66) | 1.19 (0.83, 1.69) | 1.57 (0.84, 2.94) | 1.61 (0.83, 3.12) | 0.96 (0.60, 1.53) | 0.73 (0.43, 1.23) |
| Cerebrovascular disease | 1.75 (0.64, 4.76) | 1.52 (0.44, 5.23) | 1.14 (0.60, 2.18) | 1.03 (0.49, 2.15) | 1.42 (0.49, 4.17) | 1.28 (0.38, 4.26) | **4.33 (2.31, 8.12)** | **3.80 (1.95, 7.39)** |
| Peripheral vascular  disease | 1.01 (0.40, 2.55) |  | 0.94 (0.54, 1.66) |  | 0.44 (0.06, 3.24) |  | 1.70 (0.79, 3.67) |  |
| Severe Liver disease | 1.63 (0.21, 12.93) |  | NA |  |  |  | 1.22 (0.16, 9.62) |  |
| Rheumatic disease | 1.40 (0.42, 4.67) |  | 1.50 (0.72, 3.13) |  | 0.80 (0.11, 5.96) |  | 1.80 (0.68, 4.77) |  |
| Coagulopathy | 1.94 (0.83, 4.52) | 1.66 (0.65, 4.26) | **4.47 (2.62, 7.63)** | **4.18 (2.27, 7.70)** | 2.33 (0.96, 5.65) | 1.79 (0.71, 4.49) | **3.17 (1.59, 6.34)** | 1.71 (0.68, 4.32) |
| Any malignancy | 2.03 (0.59, 6.98) |  | 1.02 (0.44, 2.37) |  | 1.13 (0.15, 8.33) |  | 0.95 (0.22, 4.11) |  |
| **Fracture type** |  |  |  |  |  |  |  |  |
| With flail chest | **2.20 (1.58, 3.08)** | **2.09 (1.48, 2.97)** | **2.02 (1.66, 2.47)** | **1.75 (1.42, 2.16)** | **2.98 (1.94, 4.59)** | **2.79 (1.79, 4.36)** | **2.23 (1.66, 3.00)** | **2.12 (1.52, 2.97)** |
| Without flail chest | Ref. | Ref. | Ref. | Ref. | Ref. | Ref. | Ref. | Ref. |
| **Pneumothorax** |  |  |  |  |  |  |  |  |
| No | Ref. |  | Ref. |  | Ref. |  | Ref. |  |
| Yes | 1.74 (0.96, 3.16) |  | 0.85 (0.53, 1.37) |  | **2.03 (1.03, 4.02)** |  | 0.81 (0.37, 1.76) |  |
| **ISS** |  |  |  |  |  |  |  |  |
| <9 | Ref. | Ref. | Ref. | Ref. | Ref. | Ref. | Ref. | Ref. |
| ≥9 | 2.19 (0.92, 5.22) | 1.61 (0.65, 4.00) | **4.73 (2.45, 9.15)** | **3.96 (2.00, 7.87)** | 2.45 (0.85, 7.03) | 1.73 (0.58, 5.10) | **12.74 (10.28, 15.79)** | **8.31 (6.23, 11.07)** |
| **Hospital bed size** |  |  |  |  |  |  |  |  |
| Small | 0.57 (0.26, 1.24) | 0.60 (0.26, 1.35) | 0.75 (0.53, 1.06) | **0.67 (0.46, 0.98)** | 1.49 (0.73, 3.04) | 1.36 (0.64, 2.87) | **0.61 (0.38, 0.98)** | **0.51 (0.32, 0.82)** |
| Medium | 0.74 (0.48, 1.12) | 0.79 (0.52, 1.20) | **0.73 (0.56, 0.94)** | **0.70 (0.54, 0.91)** | **0.48 (0.26, 0.90)** | **0.49 (0.25, 0.93)** | 0.91 (0.60, 1.39) | 0.90 (0.57, 1.44) |
| Large | Ref. | Ref. | Ref. | Ref. | Ref. | Ref. | Ref. | Ref. |
| **Hospital location/teaching status** | |  |  |  |  |  |  |  |
| Rural | **0.30 (0.25, 0.36)** | **0.37 (0.29, 0.47)** | **0.27 (0.24, 0.31)** | **0.26 (0.21, 0.31)** | 0.56 (0.07, 4.31) | 0.61 (0.08, 4.58) | **0.47 (0.40, 0.55)** | **0.60 (0.46, 0.78)** |
| Urban nonteaching | 0.62 (0.35, 1.08) | 0.64 (0.36, 1.13) | **0.66 (0.51, 0.85)** | **0.60 (0.46, 0.78)** | 0.65 (0.38, 1.09) | 0.67 (0.38, 1.16) | 0.69 (0.44, 1.08) | 0.80 (0.50, 1.29) |
| Urban teaching | Ref. | Ref. | Ref. | Ref. | Ref. | Ref. | Ref. | Ref. |
| **Hospital region** |  |  |  |  |  |  |  |  |
| Northeast | Ref. | Ref. | Ref. | Ref. | Ref. | Ref. | Ref. | Ref. |
| South | 0.96 (0.55, 1.69) | 0.92 (0.52, 1.63) | **1.56 (1.11, 2.19)** | **1.45 (1.00, 2.10)** | 1.03 (0.51, 2.06) | 1.05 (0.52, 2.10) | 1.06 (0.68, 1.65) | 0.77 (0.45, 1.30) |
| Midwest | 1.07 (0.63, 1.84) | 1.12 (0.63, 1.98) | **1.42 (1.03, 1.97)** | 1.39 (0.98, 1.98) | 1.37 (0.69, 2.75) | 1.62 (0.81, 3.22) | 0.81 (0.53, 1.24) | 0.77 (0.45, 1.32) |
| West | 1.56 (0.79, 3.06) | 0.73 (0.41, 1.31) | 1.29 (0.92, 1.79) | 1.26 (0.88, 1.82) | 0.99 (0.48, 2.05) | 1.02 (0.48, 2.16) | **0.54 (0.33, 0.88)** | **0.50 (0.29, 0.86)** |

Abbreviations: VTE, venous thromboembolism; AKI, acute kidney injury; HMO, health maintenance organization; CKD, chronic kidney disease; COPD, chronic obstructive pulmonary disease; ISS, injury severity score; NA, not applicable; Ref, reference; aOR, adjusted odd ratio; CI, confidence interval.

Significant values are shown in bold.

Variables that were significant in univariate regression were adjusted in multivariable models.

**Supplementary Table S4. Associations between obesity status and adverse events. (cont.)**

| **Variables** | **AMI** | | **CVA** | | **ARDS/respiratory failure** | | **Mechanical ventilation ≥ 96 hours** | |
| --- | --- | --- | --- | --- | --- | --- | --- | --- |
|  | **Univariate** | **Multivariate** | **Univariate** | **Multivariate** | **Univariate** | **Multivariate** | **Univariate** | **Multivariate** |
|  | **OR (95% CI)** | **aOR (95% CI)** | **OR (95% CI)** | **aOR (95% CI)** | **OR (95% CI)** | **aOR (95% CI)** | **OR (95% CI)** | **aOR (95% CI)** |
| **Morbidly obese**  **(>40 kg/m^2^)** | NA | NA | 1.47 (0.94, 2.30) | 1.36 (0.82, 2.26) | **1.72 (1.15, 2.56)** | 1.50 (0.96, 2.34) | **2.33 (1.48, 3.66)** | **2.14 (1.28, 3.58)** |
| **Obese**  **(30 ≤ BMI ≤40 kg/m^2^)** | 1.35 (0.17, 10.67) | 1.65 (0.22, 12.46) | 1.04 (0.69, 1.56) | 1.11 (0.72, 1.70) | 0.99 (0.67, 1.45) | 1.01 (0.67, 1.55) | 0.88 (0.53, 1.46) | 0.84 (0.47, 1.48) |
| **Normal weight**  **(19 ≤ BMI <30 kg/m^2^)** | Ref. | Ref. | Ref. | Ref. | Ref. | Ref. | Ref. | Ref. |
| **Age, years** |  |  |  |  |  |  |  |  |
| 20-39 | Ref. | Ref. | Ref. | Ref. | Ref. | Ref. | Ref. | Ref. |
| 40-59 | 0.35 (0.02, 5.68) | 0.29 (0.02, 5.33) | 0.92 (0.68, 1.26) | 0.85 (0.61, 1.19) | 0.99 (0.74, 1.33) | 0.94 (0.68, 1.29) | 1.05 (0.73, 1.51) | 1.03 (0.71, 1.51) |
| 60+ | 3.28 (0.41, 25.95) | 2.06 (0.24, 17.91) | 0.94 (0.69, 1.29) | 0.81 (0.57, 1.15) | 0.99 (0.74, 1.33) | 0.83 (0.59, 1.16) | 1.12 (0.77, 1.63) | 1.02 (0.68, 1.53) |
| **Sex** |  |  |  |  |  |  |  |  |
| Male | 4.15 (0.53, 32.31) |  | 0.99 (0.78, 1.26) |  | 0.84 (0.67, 1.04) |  | 1.04 (0.79, 1.38) |  |
| Female | Ref. |  | Ref. |  | Ref. |  | Ref. |  |
| **Insurance status** |  |  |  |  |  |  |  |  |
| Medicare/Medicaid | Ref. |  | Ref. |  | Ref. |  | Ref. |  |
| Private including HMO | **0.19 (0.04, 0.92)** |  | 1.16 (0.91, 1.47) |  | 1.20 (0.96, 1.50) |  | 1.05 (0.79, 1.38) |  |
| Self-pay/no-charge/other | 0.52 (0.11, 2.52) |  | 1.27 (0.96, 1.68) |  | 1.17 (0.90, 1.52) |  | 1.21 (0.88, 1.67) |  |
| **Household income** |  |  |  |  |  |  |  |  |
| Quartile1 | 0.90 (0.22, 3.61) | 0.64 (0.18, 2.35) | **1.89 (1.37, 2.61)** | **1.63 (1.14, 2.34)** | **1.93 (1.43, 2.62)** | **1.80 (1.28, 2.54)** | **1.67 (1.18, 2.37)** | **1.62 (1.11, 2.38)** |
| Quartile2 | 0.43 (0.08, 2.34) | 0.30 (0.05, 1.96) | **1.57 (1.14, 2.16)** | **1.40 (1.00, 1.96)** | **1.70 (1.26, 2.28)** | **1.57 (1.14, 2.16)** | 1.23 (0.86, 1.76) | 1.11 (0.75, 1.64) |
| Quartile3 | 0.43 (0.08, 2.38) | 0.35 (0.09, 1.37) | **1.53 (1.12, 2.10)** | 1.35 (0.96, 1.89) | **1.71 (1.27, 2.28)** | **1.58 (1.16, 2.17)** | 1.19 (0.85, 1.67) | 1.09 (0.76, 1.58) |
| Quartile4 | Ref. | Ref. | Ref. | Ref. | Ref. | Ref. | Ref. | Ref. |
| **Smoking** |  |  |  |  |  |  |  |  |
| No | Ref. |  | Ref. |  | Ref. |  | Ref. |  |
| Yes | 0.37 (0.08, 1.70) |  | **0.64 (0.51, 0.81)** |  | **0.68 (0.55, 0.84)** |  | **0.64 (0.50, 0.84)** |  |
| **Major comorbidities** |  |  |  |  |  |  |  |  |
| Diabetes | 1.80 (0.49, 6.71) |  | 0.99 (0.74, 1.32) |  | 1.00 (0.77, 1.31) |  | 1.09 (0.80, 1.50) |  |
| Hypertension | 3.11 (0.93, 10.43) |  | 1.02 (0.84, 1.26) |  | 0.97 (0.80, 1.17) |  | **0.78 (0.62, 0.99)** |  |
| CKD | 2.31 (0.29, 18.23) | 0.81 (0.10, 6.49) | 1.31 (0.80, 2.16) | 1.19 (0.63, 2.22) | 1.44 (0.91, 2.27) | 1.25 (0.69, 2.29) | 0.98 (0.54, 1.78) | 0.58 (0.27, 1.25) |
| Ischemic heart disease | **47.15 (10.13, 219.33** |  | 0.86 (0.62, 1.19) |  | 0.89 (0.66, 1.20) |  | **0.68 (0.46, 1.00)** |  |
| Congestive heart failure | **7.78 (2.05, 29.54)** | **4.83 (1.37, 16.96)** | 0.77 (0.44, 1.35) | 0.56 (0.30, 1.05) | 1.01 (0.61, 1.67) | 0.72 (0.41, 1.27) | 1.07 (0.59, 1.97) | 0.74 (0.36, 1.51) |
| Atrial fibrillation | 3.69 (0.99, 13.76) | 1.55 (0.36, 6.67) | **1.56 (1.11, 2.18)** | **1.68 (1.10, 2.57)** | **1.85 (1.35, 2.53)** | **2.01 (1.36, 2.97)** | **1.87 (1.29, 2.71)** | **1.72 (1.10, 2.71)** |
| Anemia | 2.96 (0.37, 23.43) |  | **0.18 (0.07, 0.48)** |  | 0.63 (0.35, 1.16) |  | 1.05 (0.52, 2.14) |  |
| COPD | 1.84 (0.39, 8.59) | 2.45 (0.53, 11.41) | **4.36 (3.27, 5.81)** | **4.72 (3.47, 6.42)** | **3.67 (2.76, 4.88)** | **3.89 (2.87, 5.27)** | **2.30 (1.66, 3.20)** | **2.48 (1.74, 3.53)** |
| Cerebrovascular disease | **11.68 (2.44, 55.94)** | **8.98 (1.85, 43.64)** | **4.16 (2.18, 7.94)** | **4.81 (2.24, 10.29)** | **2.12 (1.06, 4.27)** | **2.31 (1.01, 5.30)** | **2.17 (1.02, 4.63)** | 2.17 (0.94, 5.03) |
| Peripheral vascular disease | 3.21 (0.39, 26.35) |  | 0.81 (0.44, 1.49) |  | 1.09 (0.63, 1.88) |  | 0.87 (0.44, 1.71) |  |
| Severe Liver disease | NA |  | 1.04 (0.30, 3.59) |  | 1.46 (0.43, 4.96) |  | 3.10 (0.91, 10.54) |  |
| Rheumatic disease | NA |  | 0.49 (0.17, 1.41) |  | 0.64 (0.26, 1.56) |  | 0.59 (0.18, 1.98) |  |
| Coagulopathy | 3.78 (0.48, 30.12) | 1.49 (0.22, 9.94) | **0.07 (0.01, 0.52)** | **0.07 (0.01, 0.44)** | **0.57 (0.34, 0.94)** | **0.54 (0.30, 0.95)** | **2.37 (1.36, 4.10)** | **2.34 (1.21, 4.51)** |
| Any malignancy | NA |  | 0.55 (0.16, 1.86) |  | 0.61 (0.20, 1.81) |  | 0.27 (0.04, 1.98) |  |
| **Fracture type** |  |  |  |  |  |  |  |  |
| With flail chest | **3.37 (1.01, 11.19)** | 3.68 (0.89, 15.27) | **2.13 (1.73, 2.63)** | **2.08 (1.66, 2.60)** | **2.29 (1.88, 2.80)** | **2.21 (1.78, 2.74)** | **3.25 (2.56, 4.14)** | **2.99 (2.32, 3.86)** |
| Without flail chest |  |  |  |  |  |  |  |  |
| **Pneumothorax** |  |  |  |  |  |  |  |  |
| No | Ref. |  | Ref. |  | Ref. |  | Ref. |  |
| Yes | 1.49 (0.19, 11.80) |  | 1.43 (0.92, 2.24) |  | 1.22 (0.79, 1.89) |  | 1.05 (0.62, 1.78) |  |
| **ISS** |  |  |  |  |  |  |  |  |
| <9 | Ref. | Ref. | Ref. | Ref. | Ref. | Ref. | Ref. | Ref. |
| ≥9 | NA | NA | **6.76 (2.75, 16.64)** | **5.30 (2.11, 13.34)** | **4.08 (2.12, 7.88)** | **3.07 (1.52, 6.21)** | **10.54 (2.58, 43.11)** | **6.99 (1.71, 28.58)** |
| **Hospital bed size** |  |  |  |  |  |  |  |  |
| Small | NA | NA | 0.83 (0.59, 1.16) | 0.85 (0.58, 1.23) | 0.95 (0.70, 1.28) | 0.96 (0.69, 1.35) | 0.99 (0.63, 1.57) | **1.04 (0.64, 1.70)** |
| Medium | 1.31 (0.35, 4.92) | 1.47 (0.33, 6.64) | **0.66 (0.51, 0.86)** | **0.64 (0.48, 0.86)** | **0.69 (0.55, 0.86)** | **0.70 (0.54, 0.90)** | **0.67 (0.51, 0.88)** | **0.67 (0.50, 0.90)** |
| Large | Ref. | Ref. | Ref. | Ref. | Ref. | Ref. | Ref. | Ref. |
| **Hospital location/teaching status** |  |  |  |  |  |  |  |  |
| Rural | NA | NA | **0.38 (0.34, 0.42)** | **0.40 (0.32, 0.50)** | **0.47 (0.31, 0.71)** | **0.42 (0.25, 0.73)** | 0.45 (0.16, 1.24) | 0.49 (0.18, 1.34) |
| Urban nonteaching | 0.84 (0.19, 3.75) | 0.85 (0.16, 4.39) | 0.85 (0.65, 1.11) | **0.75 (0.58, 0.99)** | 0.90 (0.71, 1.15) | 0.84 (0.66, 1.07) | 0.84 (0.62, 1.15) | 0.75 (0.54, 1.05) |
| Urban teaching | Ref. | Ref. | Ref. | Ref. | Ref. | Ref. | Ref. | Ref. |
| **Hospital region** |  |  |  |  |  |  |  |  |
| Northeast | Ref. | Ref. | Ref. | Ref. | Ref. | Ref. | Ref. | Ref. |
| South | 2.13 (0.23, 19.31) | 2.90 (0.22, 38.08) | 1.03 (0.71, 1.48) | 1.00 (0.67, 1.50) | 0.98 (0.69, 1.39) | 0.89 (0.60, 1.31) | 1.33 (0.90, 1.96) | 1.23 (0.80, 1.89) |
| Midwest | 2.38 (0.27, 20.85) | 3.59 (0.27, 47.00) | 1.09 (0.78, 1.51) | 1.11 (0.76, 1.63) | 1.02 (0.74, 1.41) | 0.97 (0.67, 1.41) | 1.22 (0.85, 1.75) | 1.22 (0.83, 1.82) |
| West | 0.92 (0.08, 10.50) | 1.03 (0.06, 18.89) | 0.94 (0.67, 1.32) | 1.04 (0.71, 1.53) | 0.92 (0.66, 1.29) | 0.96 (0.66, 1.38) | 1.13 (0.77, 1.64) | 1.16 (0.77, 1.74) |

Abbreviations: AMI, acute myocardial infarction; CVA, cerebrovascular accident; HMO, health maintenance organization; CKD, chronic kidney disease; COPD, chronic obstructive pulmonary disease; ISS, injury severity score; NA, not applicable; Ref, reference; aOR, adjusted odd ratio; CI, confidence interval.

Significant values are shown in bold.

Variables that were significant in univariate regression were adjusted in multivariable models.

**Supplementary Table S5. Associations between obesity status and adverse events, stratified by with and without flail chest.**

| **Subgroup** | Tracheostomy | | Pneumonia | | VTE | |
| --- | --- | --- | --- | --- | --- | --- |
|  | aOR (95% CI) | p-value | aOR (95% CI) | p-value | aOR (95% CI) | p-value |
| **With flail chest** |  |  |  |  |  |  |
| Morbidly obese  (>40 kg/m^2^) | 2.13 (1.05, 4.32) | **0.036** | 0.87 (0.39, 1.97) | 0.740 | 1.91 (0.81, 4.52) | 0.138 |
| Obese  (30 ≤ BMI ≤40 kg/m^2^) | 0.38 (0.08, 1.81) | 0.222 | 0.98 (0.41, 2.33) | 0.959 | 1.30 (0.42, 4.05) | 0.652 |
| Normal weight  (19 ≤ BMI <30 kg/m^2^) | Ref. |  | Ref. |  | Ref. |  |
| **Without flail chest** |  |  |  |  |  |  |
| Morbidly obese  (>40 kg/m^2^) | NA |  | 1.70 (0.71, 4.07) | 0.229 | 1.80 (0.58, 5.57) | 0.309 |
| Obese  (30 ≤ BMI ≤40 kg/m^2^) | 0.50 (0.16, 1.50) | 0.213 | 1.03 (0.48, 2.18) | 0.945 | 1.55 (0.46, 5.16) | 0.476 |
| Normal weight  (19 ≤ BMI <30 kg/m^2^) | Ref. |  | Ref. |  | Ref. |  |

| **Subgroup** | ARDS/respiratory failure | | Mechanical ventilation ≥ 96 hours | |
| --- | --- | --- | --- | --- |
|  | aOR (95% CI) | p-value | aOR (95% CI) | p-value |
| **With flail chest** |  |  |  |  |
| Morbidly obese  (>40 kg/m^2^) | 2.01 (1.09, 3.70) | **0.024** | 2.80 (1.47, 5.32) | **0.002** |
| Obese  (30 ≤ BMI ≤40 kg/m^2^) | 0.58 (0.30, 1.13) | 0.107 | 0.55 (0.25, 1.22) | 0.137 |
| Normal weight  (19 ≤ BMI <30 kg/m^2^) | Ref. |  | Ref. |  |
| **Without flail chest** |  |  |  |  |
| Morbidly obese  (>40 kg/m^2^) | 1.17 (0.60, 2.26) | 0.647 | 1.64 (0.71, 3.79) | 0.249 |
| Obese  (30 ≤ BMI ≤40 kg/m^2^) | 1.36 (0.84, 2.20) | 0.203 | 1.05 (0.53, 2.06) | 0.891 |
| Normal weight  (19 ≤ BMI <30 kg/m^2^) | Ref. |  | Ref. |  |

Adjusted for age group, household income, smoking, CKD, congestive heart failure, atrial fibrillation, COPD, cerebrovascular disease, coagulopathy, ISS, hospital bed size, hospital location/teaching status and hospital region

P-value < 0.05 are showed in bold

Abbreviations: AKI, acute kidney injury; ARDS, acute respiratory distress syndrome; VTE, venous thromboembolism; CKD, chronic kidney disease; COPD, chronic obstruction pulmonary disease; ISS, injury severity score; Ref, reference; aOR, adjusted odd ratio; CI, confidence interval.
